# Response of Chinese cabbage (*Brassica rapa* subsp. *pekinensis*) to bacterial soft rot infection by change of soil microbial community in root zone

**DOI:** 10.3389/fmicb.2024.1401896

**Published:** 2024-05-09

**Authors:** Xuqing Li, Xiaoxu Ren, Ezzeldin Ibrahim, Haimin Kong, Maofeng Wang, Jiaojiao Xia, Hong Wang, Linfei Shou, Tiefeng Zhou, Bin Li, Jianli Yan

**Affiliations:** ^1^Institute of Vegetable, Hangzhou Academy of Agricultural Sciences, Hangzhou, China; ^2^State Key Laboratory of Rice Biology and Breeding, Ministry of Agriculture Key Laboratory of Molecular Biology of Crop Pathogens and Insects, Key Laboratory of Biology of Crop Pathogens and Insects of Zhejiang Province, Institute of Biotechnology, Zhejiang University, Hangzhou, China; ^3^Zhejiang Management Station of Cropland Quality and Fertilizer, Hangzhou, China; ^4^Agricultural Office of Daciyan Town, Jiande, China; ^5^Soil Fertilizer and Plant Protection Station in Qingtian County, Qingtian, Zhejiang, China; ^6^Station for the Plant Protection and Quarantine and Control of Agrochemicals of Zhejiang Province, Hangzhou, China

**Keywords:** Chinese cabbage, bacterial soft rot, healthy, diseased, microbial community, plant-growth-promoting microbe

## Abstract

Chinese cabbage, scientifically known as *Brassica rapa* subsp*. pekinensis,* is a highly popular vegetable in China for its delectable taste. However, the occurrence of bacterial soft rot disease poses a significant threat to its growth and overall development. Consequently, this study aimed to explore the defense mechanisms employed by Chinese cabbage against bacterial soft rot disease. Specifically, the investigation focused on understanding the relationship between the disease and the microbial communities present in the soil surrounding the roots of Chinese cabbage. Significant disparities were observed in the composition of microbial communities present in the root-zone soil of healthy Chinese cabbage plants compared to those affected by *Pectobacterium brasiliense*-caused soft rot disease. The analysis of 16S rRNA gene high-throughput sequencing results revealed a lower abundance of Proteobacteria (8.39%), Acidobacteriot (0.85), *Sphingomonas* (3.51%), and *Vicinamibacteraceae* (1.48%), whereas Firmicutes (113.76%), Bacteroidota (8.71%), Chloroflexi (4.89%), Actinobacteriota (1.71%), A4b (15.52%), *Vicinamibacterales* (1.62%), and *Gemmatimonadaceae* (1.35%) were more prevalent in healthy plant soils. Similarly, the analysis of ITS gene high-throughput sequencing results indicated a reduced occurrence of Chytridiomycota (23.58%), Basidiomycota (21.80%), *Plectosphaerella* (86.22%), and *Agaricomycetes* (22.57%) in healthy soils. In comparison, Mortierellomycota (50.72%), Ascomycota (31.22%), *Podospora* (485.08%), and *Mortierella* (51.59%) were more abundant in healthy plant soils. In addition, a total of 15 bacterial strains were isolated from the root-zone soil of diseased Chinese cabbage plants. These isolated strains demonstrated the ability to fix nitrogen (with the exception of ZT20, ZT26, ZT41, ZT45, and ZT61), produce siderophores and indole acetic acid (IAA), and solubilize phosphate. Notably, ZT14 (*Citrobacter freundii*), ZT33 (*Enterobacter cloacae*), ZT41 (*Myroides odoratimimus*), ZT52 (*Bacillus paramycoides*), ZT58 (*Klebsiella pasteurii*), ZT45 (*Klebsiella aerogenes*), and ZT32 (*Pseudomonas putida*) exhibited significant growth-promoting effects as determined by the plant growth promotion (PGP) tests. Consequently, this investigation not only confirmed the presence of the soft rot pathogen in Chinese cabbage plants in Hangzhou, China, but also advanced our understanding of the defense mechanisms employed by Chinese cabbage to combat soft rot-induced stress. Additionally, it identified promising plant-growth-promoting microbes (PGPMs) that could be utilized in the future to enhance the Chinese cabbage industry.

## 1 Introduction

Chinese cabbage, scientifically known as *Brassica rapa* subsp. *pekinensis*, has originated in China and eastern Asia since the fifth century (Kalloo and Rana, 1993). It is one of the largest cultivated and consumed vegetables in China these days and is becoming more and more popular worldwide (Li J. et al., 2022) due to its richness in various minerals, vitamins, carotenoids, flavonols, and glucosinolate (Podsedek, 2007; Lai et al., 2010; Lee et al., 2019). However, bacterial soft rot disease, one of the most prevalent diseases affecting Chinese cabbage, poses a significant challenge to its production and supply (Alvarado et al., 2011; Liu et al., 2019). Extensive research has been conducted to address this issue and ensure stable Chinese cabbage yields. The soft rot disease in Chinese cabbage is commonly associated with the presence of *Pectobacterium carotovorum*, which encompasses *P. carotovorum* subsp. *carotovorum* (*Pcc*), *P. carotovorum* subsp. *brasiliense* (*Pcb*), and *P. carotovorum* subsp. *odoriferum* (*Pco*; Pritchard et al., 2016; Li et al., 2020). These microorganisms are thought to be the main causes of Chinese cabbage soft rot disease. Quick detection and identification of *P. carotovorum* ssp. by subspecies-specific PCR based on the *pmr*A gene, different isolation and growth conditions between different subspecies, and 16S rRNA gene sequencing at the genus or species level is done (Naum et al., 2008; Kettani-Halabi et al., 2013; Czajkowski et al., 2014).

Chemical bactericides have been widely employed to manage the prevalence of soft rot bacteria efficiently. However, their utilization has become restricted due to their high expenses and potential toxicity to humans. As an alternative, the focus has shifted toward biological control methods, emphasizing the application of soil microbial agents to combat pathogens. This approach has garnered increased attention as a safer and more sustainable means of disease management (Abd-EI-Khair et al., 2021; Wang et al., 2023). Where, *Bacillus* spp. demonstrated effective activity against *Pecarotovorum* subsp. *carotovorum* in *in vitro* tests, while *Pseudomonas* spp. exhibited control over soft rot disease in valerian rhizomes, also displaying the ability to enhance root weights. Furthermore, *Trichoderma* spp. demonstrated effective control of soft rot disease in both *in vitro* and *in vivo* tests (Ghods-Alavi et al., 2012; Issazadeh et al., 2012; Abd-EI-Khair et al., 2021). Previous studies have consistently shown a clear relationship between the abundance and diversity of soil microbes and the occurrence of soil-borne diseases (Jia et al., 2023). The soil microbial community structure and composition plays a critical role in maintaining ecosystem resilience and sustainability, including the suppression of pathogens (Wagg et al., 2014), which expanded traditional plant pathogen interaction research (He et al., 2023; Liu et al., 2023; Tao et al., 2023). In recent years, high-throughput sequencing technology has emerged as a powerful tool for rapidly and accurately assessing the diversity and composition of microbial communities in diverse sample types. This technology enables comprehensive and efficient analysis of microbial populations, facilitating a deeper understanding of their roles and interactions in soil ecosystems (Reuter et al., 2015).

Additionally, soil microorganisms—especially bacteria and fungi that promote plant growth—play a critical role in agriculture ecosystems. Plant Growth Promoting Microbes (PGPMs) can settle in plant roots and help their hosts in a number of ways, including improving plant nutrition uptake through fixation, mobilization, mineralization, and solubilization of nutrients; the production of siderophores and antibiotics; the release of indole acetic acid (IAA) and gibberellin hormones; alleviating biotic and abiotic stresses; enhancing plant growth; and improving plant productivity and food quality (Etesami, 2020; Suman et al., 2022). *Acidothiobacillus*, *Azospirillum*, *Azotobacter*, *Bacillus*, *Beauveria*, *Burkholderia*, *Enterobacter*, *Clostridium*, *Flavobacterium*, *Klebsiella*, *Frankia*, *Rhizobium*, *Pantoea*, *Pseudomonas*, *Serratia*, *Streptomyces*, *Trichoderma*, *Aspergillus*, and *Penicillium* have invaluable uses in agriculture owing to their cost-effectiveness, environmental friendliness, and improved plant yield (Abhilash et al., 2016; Oosten et al., 2017; Raimi et al., 2017; Gouda et al., 2018). The success and effective utilization of PGPMs are key and important for promoting sustainable agriculture, which can achieve a safe environment and, in turn, positively influence human health (Suman et al., 2022).

In this study, we collected root-zone soil samples from both healthy and diseased Chinese cabbage plants. The primary objectives were to compare the microbial community structure and diversity between healthy and diseased plants using high-throughput sequencing and to isolate PGPMs. By doing so, we aimed to establish a clearer understanding of the relationship between soft rot occurrence in Chinese cabbage and the associated microbial community, and further to determine the soil microbial community composition mediated defense mechanisms employed by Chinese cabbage against bacterial soft rot disease. This research provides a theoretical foundation for disease control strategies and offers practical guidance for Chinese cabbage production in the future. Specifically, our study aims to identify key pathogens, investigate differences in the soil microbial community structure between healthy and diseased Chinese cabbage plants, and isolate PGPMs capable of thriving under conditions of soft rot stress.

## 2 Materials and methods

### 2.1 Sample collection

On 7 October 2023, Chinese cabbage plants exhibiting clear symptoms of soft rot were sampled from a field located at the Zhijiang Base of Hangzhou Academy of Agricultural Sciences (30°9′12″N; 119°5′36″E; 12 m above sea level) in Hangzhou, China, to isolate bacterial pathogens responsible for soft rot. Additionally, 12 root-zone soil samples were collected for the isolation of PGPMs and comparative analysis of the microbial community structure and composition. Six soil samples were collected from the root zones of plants affected by soft rot, while the remaining six samples were obtained from healthy plants. Soil samples were collected from the top 5–20 cm layer using a sterile stainless-steel shovel to ensure aseptic conditions. The soil in the sampled area had a pH of 7.77, an organic matter content of 0.95%, an alkaline hydrolysis N content of 43.87 mg/kg, an available P content of 83.83 mg/kg, and an available K content of 436.41 mg/kg. Each soil sample was carefully packed into a sterile bag and stored in an ice box during transportation to the laboratory for further analysis.

### 2.2 Isolation and identification of pathogenic strains

To isolate pathogenic bacteria from Chinese cabbage stems exhibiting soft rot symptoms, the method described by Lee et al. (2019) was used. Specifically, the junction areas of the stems (the area between infected or not by the pathogen) were cut into 30 × 30 mm pieces. These stem pieces were then subjected to a series of sterilization steps, which involved immersing them in 75% alcohol for 45 s, followed by rinsing in sterile distilled water three times. Subsequently, the stem pieces were crushed in 2 mL of sterile distilled water, and a 100 μL suspension was smeared onto Luria-Bertani (LB) agar medium, consisting of tryptone (10 g), yeast extract (5 g), NaCl (10 g), agar (20 g), and distilled water (1,000 mL) with a pH of 7.2. The agar plates were then incubated at 28°C for 24 h. Various bacterial colonies were selected and purified by streaking them onto LB agar medium three times. The purified colonies were subsequently used for pathogenicity testing.

For the pathogenicity test, a single colony of each bacterial strain was picked and suspended in 5 mL of LB broth. The suspension was incubated in a shaker at 28°C for 16 h. After reaching a cell density of approximately 10^6^ colony-forming units (CFU) per mL, 10 μL of the cell suspension for each test strain was injected into 60 × 60 mm healthy Chinese cabbage stems placed in 90 mm Petri dishes containing wet filter papers. The Petri dishes were then incubated at 28°C for 48 h. The ones injected with 10 μL of sterile water were used as the control. The strains that caused the same symptoms as before were identified as pathogens.

Similarly, to isolate plant-growth-promoting bacteria (PGPB) from the root-zone soil of diseased Chinese cabbage, the following procedure was followed: Twenty grams of each soil sample were mixed with 180 mL of distilled water (ddH_2_O) separately and shaken for 30 min at 120 rpm at a temperature of 4°C. The resulting suspension was then subjected to a 10-fold dilution. Subsequently, 100 μL of the 10^4^ dilution was spread onto LB agar medium and incubated at 28°C for 24 h. Based on the variations in morphological characteristics, distinct colonies were selected and purified on an LB agar medium. All purified bacterial strains were preserved by storing them in a 20% glycerin solution at a temperature of −40°C in an ultra-low refrigerator for future use.

To further identify the strains obtained from the previous steps, a sequence analysis of the 16S rRNA and *pmr*A genes was conducted. The methodology used for this analysis was based on the procedures described by Tomah et al. (2020) for the 16S rRNA gene and Kettani-Halabi et al. (2013) for the *pmr*A gene. In brief, the 16S rRNA genes of the isolated strains were amplified using the universal primers 27F (5′-AGAGTTTGATCCTGGCTCAG-3′) and 1492R (5′–GGTTACCTTGTTACGACTT-3′; Tomah et al., 2020), while the *pmr*A genes were amplified using the specific primers F0145 (5′-TACCCTGCAGATGAAATTATTGATTGTTGAAGAC-3′) and E2477 (5′-TACCAAGCTTTGGTTGTTCCCCTTTGGTCA-3′; Hyytiainen et al., 2003). The PCR reaction mixture (50 μL) included ddH_2_O (18 μL), 2 × Hieff® PCR Master Mix (25 μL), 10 μM forward primer (2 μL), 10 μM reverse primer (2 μL), and DNA (3 μL). The PCR was carried out using the following steps: 94°C 5 min; 94°C 30 s, 53°C (for 16S rRNA gene) or 57°C (for *pmr*A gene) 30 s, 72°C 1 min, 35 cycles; 72°C 10 min. PCR products were visualized on 1.0% agarose gels, then purified and submitted to Tsingke Biotechnology Co., Ltd. (Hangzhou, China) for sequencing in both directions. All the obtained sequences were aligned with the sequences of NCBI by nucleotide BLAST (similarity >97%) to determine the taxonomy of the strains (Shi et al., 2019).

### 2.3 Evaluation of difference in microbial community structure and composition

To assess the differences in microbial community structure and composition between healthy and diseased Chinese cabbage in the root-zone soil, the following methods were employed: A 10-gram soil sample was taken from each sample, and soil DNA extraction was performed using the E.Z.N.ATM Mag-Bind Soil DNA Kit (OMEGA, United States). Bacterial diversity was evaluated by targeting the V3-V4 region of the 16S rRNA gene, while fungal diversity was assessed by targeting the ITS1/ITS2 region. For bacterial diversity analysis, the V3-V4 region was amplified using the primer sets 341F (5′-CCTACGGGNGGCWGCAG-3′) and 805R (5′-GACTACHVGGGTATCTAATCC-3′; Wu L. Y. et al., 2015). On the other hand, for fungal diversity analysis, the ITS1/ITS2 region was amplified using the primer sets ITS1F (5′-CTTGGTCATTTAGGAAGTAA-3′) and ITS (5′-GCTGCGTTCTTCATTCGATGC-3′; Li X. Q. et al., 2022).

To make the PCR reaction mixture (30 μL), we used ddH_2_O (11 μL), 2 × Hieff® Robust PCR Master Mix (15 μL), 10 μM forward primer (1 μL) and reverse primer (1 μL), as well as DNA (2 μL). The PCR was carried out using the following steps: 95°C 3 min; 95°C 30 s, 45°C 30 s, 72°C 30 s, 5 cycles; 95°C 30 s, 55°C 30 s, 72°C 30 s, 20 cycles; 72°C 5 min. The amplicon products were purified using Hieff NGS™ DNA Selection Beads (Yeasen, China). Afterward, amplicons were pooled in equimolar amounts, and 2 × 250-bp pair-end sequencing was performed using the Illumina MiSeq system (Illumina MiSeq, United States), according to the manufacturer’s instructions.

After the sequencing process, the short Illumina reads were assembled using PEAR (v0.9.8). Quality control was performed to ensure data integrity by removing reads with a Phred33 score of less than 20 using Trimmomatic (v0.39; Bolger et al., 2014). Additionally, primers were trimmed from the reads using Cutadapt (v3.5; Martin, 2011). Raw reads underwent further processing, including quality filtering, denoising, merging, and removal of chimeric sequences, using the DADA2 algorithm (Callahan et al., 2016). The resulting clean reads were then clustered into operational taxonomic units (OTUs) based on a similarity threshold of ≥97% using Usearch (v11.0.667). To determine the taxonomic classification of the bacterial and fungal OTU representative sequences, a BLAST search was performed against the RDP Database for bacterial sequences and the UNITE fungal ITS Database for fungal sequences. This classification step allowed for the assignment of taxonomic labels to the OTUs.

### 2.4 Growth-promoting properties of isolated bacterial strains

After activating the bacterial strains on LB agar plates at 28°C for 24 h, a single colony from each strain was selected and incubated in LB broth at 28°C and 180 rpm for 18 h in a shaker. The density of the resulting cell suspension was adjusted to 10^7^ CFU per mL. Sterile filter paper disks, measuring 6 mm in diameter, were immersed in each suspension for duration of 10 s. Subsequently, the soaked disks were positioned at the center of the respective test media, including National Botanical Research Institute’s Phosphate (NBRIP), Chrome Azurol S (CAS), N-free malate (Nfb), and LB, for the evaluation of their plant growth properties. To evaluate the phosphate-solubilizing ability, the NBRIP medium was used. The NBRIP medium composition included glucose (10 g), Ca_3_ (PO_4_)_2_ (5 g), MgCl_2_•6H_2_O (10.67 g), KCl (0.2 g), MgSO_4_•7H_2_O (0.25 g), (NH_4_)_2_SO_4_ (0.1 g), agar (25 g), and distilled water (1,000 mL) with a pH value of 7.0 (Nautiyal, 1999). The test bacteria were incubated on NBRIP media at 28°C for 3 days, and the transparent halo formed around the colony was observed and measured using a Vernier caliper (CD-20APX, Mitutoyo, Japan). To assess the production of siderophores, the CAS medium was used. The CAS medium composition consisted of CAS (0.0605 g), FeCl_3_•6H_2_O (1 mM, with 10 mM HCl) (10 mL), distilled water (50 mL), HDTMA (0.0729 g), distilled water (40 mL), pipes (30.24 g), MM9 salts (10x) (100 mL), agar (15 g), distilled water (750 mL) with a pH value of 6.8, casamino acids (10%) (30 mL), glucose (20%) (10 mL), MgCl_2_ (1 M) (1 mL), and CaCl_2_ (100 mM) (1 mL; Murugappan et al., 2011). After incubating on the CAS plate at 28°C for 3 days, the orange halo around the colony was observed and measured as before. Strains that showed the ability to solubilize phosphate and produce siderophore were selected to evaluate their ability to fix nitrogen and produce IAA. To assess the nitrogen-fixing ability, the Nfb medium was used. The Nfb medium composition included KH_2_PO_4_ (0.4 g), K_2_HPO_4_ (0.1 g), MgSO_4_•7H_2_O (0.2 g), NaCl (0.1 g), CaCl_2_ (0.02 g), FeCl_3_ (0.01 g), MoO_4_Na_2_•2H_2_O (0.002 g), sodium malate (5 g), bromothymol blue (with a 0.5% alcohol solution; 5 mL), distilled water (1,000 mL), with a pH value of 7.0 (Tang et al., 2020). 10 μL from each suspension strain was added to a 10 mL tube containing 5 mL of the Nfb medium. The tubes were then incubated at 28°C and 180 rpm for 2 days in a shaker. The presence or absence of a color change from green to blue in the medium was observed to evaluate the nitrogen-fixing ability. To evaluate the IAA production ability of the bacterial strains, the cell suspension was adjusted to a density of 10^7^ CFU/mL. Subsequently, 10 μL of each test cell suspension was added to a 15-mL tube containing 5 mL of LB medium supplemented with either 0.1% or 1.0% tryptophan. The tubes were then incubated at 28°C and 180 rpm for 2 days in a shaker. After the incubation period, 1.5 mL of the cell suspension in each tube was centrifuged at 4°C and 8,000 rpm for 5 min. Following centrifugation, 500 μL of the supernatant was mixed with 2 mL of Salkowski’s reagent, which consisted of FeCl_3_ (0.5 M), H_2_SO_4_, and distilled water in a ratio of 3:60:100. The mixture was then incubated at 28°C for 20 min in the dark. The production of IAA was assessed by observing a color change from pink to red, and the optical density (OD) at 530 nm was measured using a UV–Vis spectrophotometer (Li et al., 2021).

### 2.5 Effect of PGPB strains on Chinese cabbage growth under soft rot stress

To assess the effects of PGPB strains on Chinese cabbage growth under soft rot stress, susceptible cultivar Xiayangbai seeds (procured from Nanjing Lvling Seed Industry Co., Ltd., Nanjing, China) were used (Tan and Huang, 2005). The experimental procedure involved the following steps: the seeds were sterilized by immersion in 75% alcohol for 1 min, followed by rinsing with sterile distilled water three times. Subsequently, the sterilized seeds were pre-germinated on filter paper in 90-mm dishes with sterile water at a temperature of 25°C for 24 h. The pre-germinated seeds were then sown individually in planting pots (8 cm × 8 cm × 12.5 cm) containing soil collected from the same field. The pots were placed in a greenhouse with a temperature of 25°C and 70% relative humidity. The PGPB strains were prepared as cell suspensions by incubating them in a shaker at 28°C and 180 rpm for 18 h, and the cell density was adjusted to 10^8^ CFU/mL. At the two-leaf stage, which occurred 3 days after sowing, 10 mL of the cell suspension of each tested strain was applied to the soil surrounding the Chinese cabbage plants. As a control, Chinese cabbages were irrigated with 10 mL of sterile water. Each treatment consisted of eight Chinese cabbage plants (*n* = 8), and three replicates were carried out. After 28 days of inoculation, various parameters, including leaf number, root length, fresh weight, and dry weight of the seedlings and roots, were measured using an electronic scale (TCS-50, Shanghai, China) and a Vernier caliper. To determine these measurements, the Chinese cabbage plants were carefully removed from their respective pots, and the soil was gently washed from the roots using tap water. The root length and fresh weight were determined, and the root plants were then dried in an oven at 65°C for 48 h to obtain the dry weight. The percentage of growth promotion efficacy (GPE) was calculated using the formula GPE% = (treatment − control) / control × 100% to determine the relative effect of the tested strains on Chinese cabbage growth.

### 2.6 Statistical analysis

The statistical analysis was performed using the software package SPSS (v16.0) from Chicago, United States. One-way ANOVA tests were conducted to analyze variance. Bar graphs were generated using Origin software (v2022) to visualize the OTU richness. Origin software was also used to calculate *α*-diversity indices (Chao1, Shannon) and display the results. Relative abundances (RAs) and heat maps of the dominant bacteria and fungi at the phylum and genus levels were calculated using Origin software. *β*-diversity was assessed through permutational analysis of variance (PERMANOVA) to compare soil microorganisms across samples. Principal component analysis (PCA) was performed using the R package “vegan” (v2.5–6) to visualize the results, following the methods described by Dixon (2003) and Ramette (2007). To identify biomarkers and estimate their effect size, linear discriminant analysis (LDA) effect size (LEfSe) was conducted using the OmicStudio tools.^1^ A significance threshold of alpha value <0.05 and LDA score > 2.5 (for bacteria) or 3 (for fungi) was used, as described by Segata et al. (2011).

## 3 Results

### 3.1 Isolation and identification of soft rot pathogen

**Figure 1:**
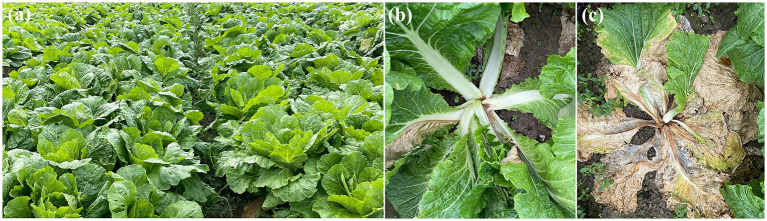
Shows the plant situation in the field **(A)**, as well as the symptoms of the disease at an early stage **(B)** and a late stage **(C)**.

**Figure 2:**
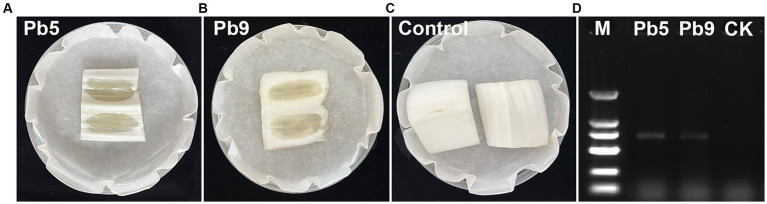
Illustrates the symptoms observed in Chinese cabbage stems inoculated with Pb5 **(A)**, Pb9 **(B)**, and water **(C)**. In addition, depicts the amplification of a specific 686 bp band in the two strains using the *pmr*A F0145 and E2477 primers **(D)**. M: 2,000 bp Marker (100, 250, 500, 750, 1,000, and 2,000 bp, respectively).

To isolate and identify the pathogen responsible for bacterial soft rot on Chinese cabbage cultivated in Hangzhou, China, several cabbage stems exhibiting clear symptoms were collected (Figure 1). A total of nine strains were isolated from cabbage stems, but only two strains (Pb5, Pb9) induced soft rot symptoms based on pathogenicity tests (Figures 2A–C). Specifically, cabbage stems inoculated with Pb5 and Pb9 displayed soft, watery, and tan-colored symptoms, while the control stems inoculated with water did not show any symptoms.

Based on the 16S rRNA gene sequences analysis, two 1,418 bp fragments were obtained, and the 16S rRNA sequence of Pb5 (PP434478) and Pb9 (PP434479) showed high similarity to *Pectobacterium carotovorum* (99.72%) and *P. brasiliense* (99.72%). To further identify the pathogen, *pmr*A gene sequence analysis was performed. Indeed, two 686 bp fragments were obtained (Figure 2D), and the *pmr*A sequences of Pb5 (PP445233) and Pb9 (PP445234) showed high similarity to *P. brasiliense* (99.70%). Therefore, it can be concluded that Pb5 and Pb9 were identified as *P. brasiliense*.

### 3.2 Differences in the root-zone soil microbial community between healthy and diseased Chinese cabbage

**Figure 3:**
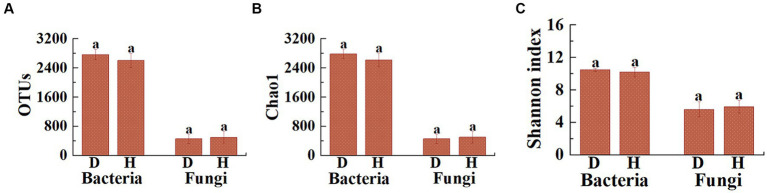
Illustrates the differences in the distribution of OTUs **(A)**, as well as the Chao1 **(B)** and Shannon **(C)** indices of microbial communities, between the root-zone soil of diseased and healthy Chinese cabbage. Statistical differences (*p* < 0.05) are indicated by different lowercase letters above the columns.

Based on the high-throughput amplicon sequencing, a total of 32,223 bacterial and 5,707 fungal OTUs were identified from 12 soil samples. Differences were observed in the number of bacterial and fungal OTUs between the diseased and healthy Chinese cabbages (Figure 3A). Indeed, the average number of bacterial OTUs in diseased Chinese cabbage was 2,767.5 (2,542 to 2,967), while in healthy Chinese cabbages it was 2,603.0 (2,363 to 2,890). This indicates that the healthy Chinese cabbage exhibited a 5.94% reduction in the number of bacterial OTUs in root-zone soils compared to the diseased ones. On the other hand, the average number of fungal OTUs in diseased Chinese cabbage is 454.0 (332 to 643), whereas in healthy Chinese cabbage, it is 497.2 (350 to 788). This suggests that the healthy Chinese cabbage showed a 9.51% increase in the number of fungal OTUs in the root-zone soils compared to the diseased ones.

Furthermore, the *α*-diversity (including Chao1 and Shannon) analysis was performed to evaluate the species richness and diversity of microbial communities in 12 soil samples (Figures 3B,C). Indeed, the average bacterial Chao1 index was 2,783.8 (2,560.9 to 2,975.1), 2,617.9 (2,382.8 to 2,904.0), and the average bacterial Shannon index was 10.50 (10.14 to 10.70), and 10.20 (9.25 to 10.63) in diseased and healthy Chinese cabbage, respectively. In general, healthy Chinese cabbage caused a 5.96 and 2.88% decrease in the bacterial Chao1 and Shannon index of root-zone soils compared to those of diseased ones, respectively. Meanwhile, the average fungal Chao1 index was 455.0 (332.2 to 647.4), 498.8 (356.0 to 790.5), and the average fungal Shannon index was 5.63 (4.76 to 6.98), and 5.94 (5.32 to 6.98) in diseased and healthy Chinese cabbage, respectively. In other words, healthy Chinese cabbage caused a 9.63% and 5.54% increase in the fungal Chao1 and Shannon index of root-zone soils, respectively, compared to diseased ones. Overall, the bacterial richness in the root-zone soil of healthy Chinese cabbage was less than that in the diseased ones, while the fungal richness in the root-zone soil of healthy Chinese cabbage was greater than that in the diseased ones, and the *α*-diversity of soil microbes in the root-zone soil of healthy and diseased Chinese cabbage did not show a significant difference.

**Figure 4:**
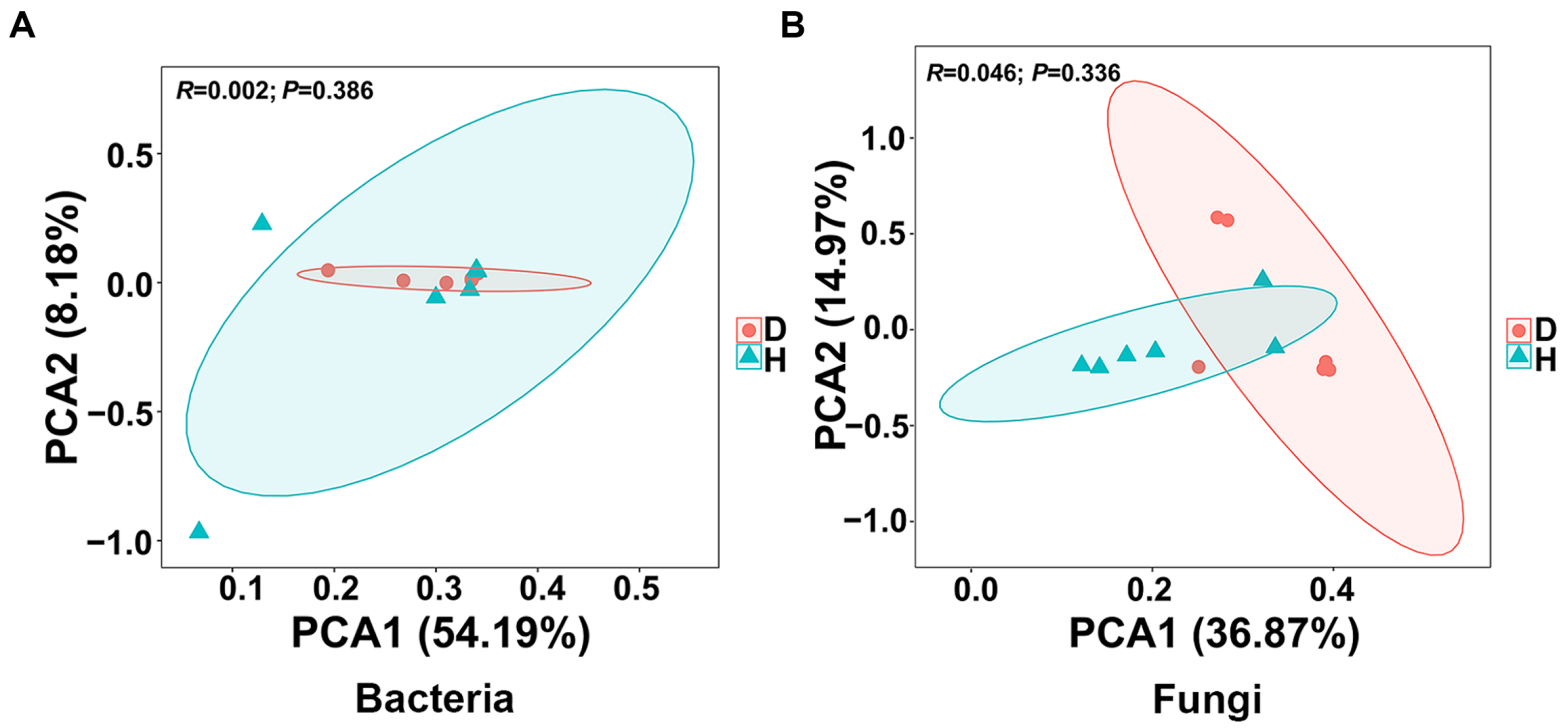
Presents the results of principal component analysis (PCA) conducted at the bacterial **(A)** and fungal **(B)** OTU level. Ellipses are included in the plot, indicating the 0.95 confidence limit.

To assess the similarity and dissimilarity in bacterial and fungal communities in the root-zone soil samples, the Bray-Curtis metric was employed to evaluate the *β*-diversity analysis and visualized by PCA. Notably, the OTU abundance from 12 soil samples of healthy and diseased Chinese cabbages exhibited distinct clustering into two groups based on the severity of soft rot. However, there was noticeable overlap observed regardless of whether the analysis focused on bacterial or fungal data (Figure 4). In the bacterial communities, the first principal component accounted for 54.19% of the variance, and the second component explained 8.18%, which showed the two principal components accounted for around 62% of the total variance. In the fungal communities, the first and second principal components accounted for 36.87% and 14.97% of the variance, respectively, which meant the two principal components accounted for around 52% of the total variance. These findings suggest that the bacterial and fungal communities in the root-zone soil of diseased and healthy Chinese cabbage were generally similar, albeit with some differences.

**Figure 5:**
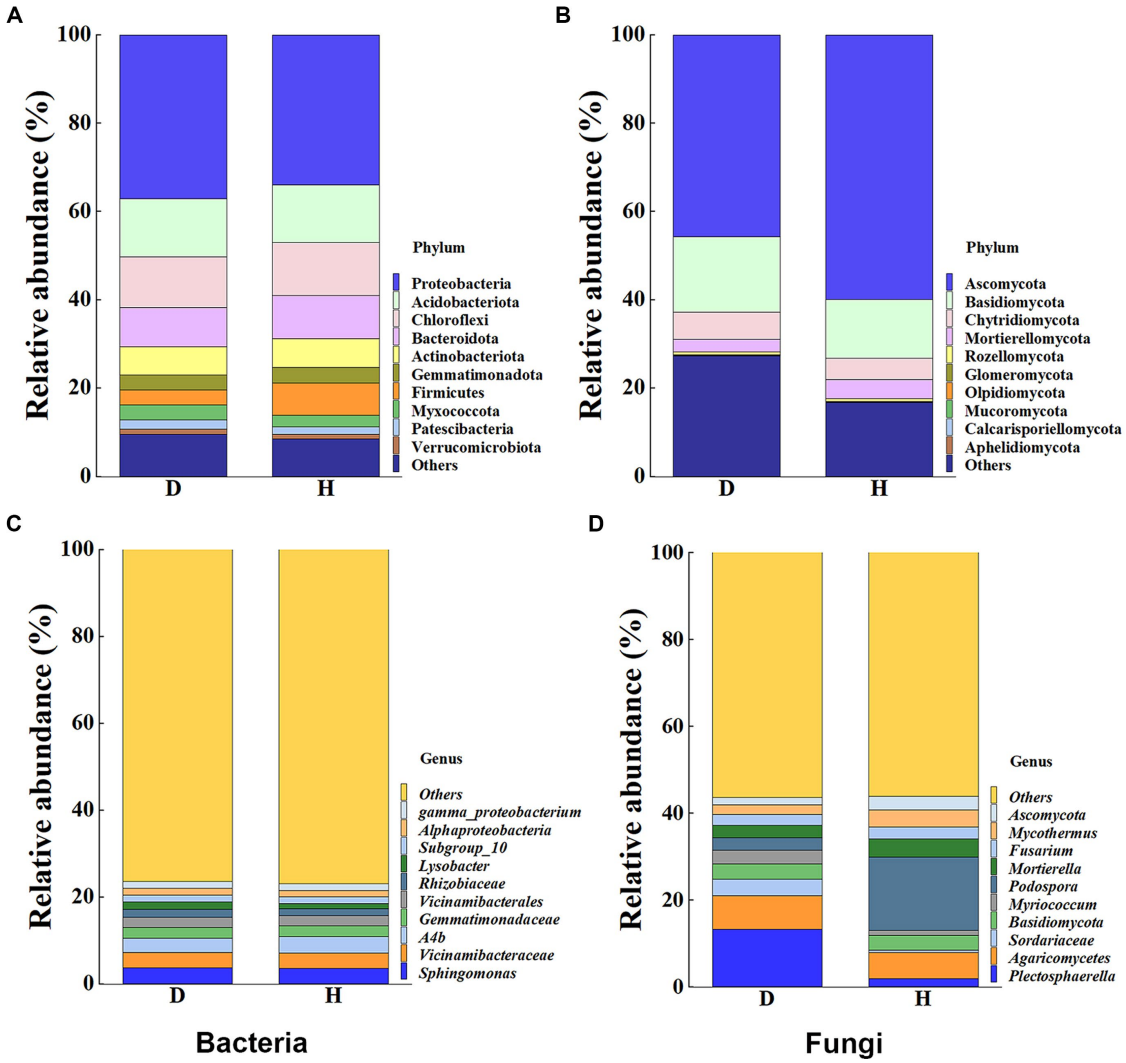
Histogram of relative abundances (RAs) at the bacterial **(A,C)** and fungal **(B,D)** phylum and genus level, respectively.

To provide a clearer representation of the disparities in bacterial and fungal communities between diseased and healthy Chinese cabbage root-zone soil, a histogram of RAs at the top 10 phylum and genus levels was carried out (Figure 5). The results indicated that the microbial community structure of diseased Chinese cabbage root-zone soil varied from that of healthy ones. In detail, in the bacterial community, Proteobacteria, Acidobacteriota, Chloroflexi, Bacteroidota, Actinobacteriota, and Firmicutes were the main phyla (Figure 5A). Compared with the diseased Chinese cabbage root-zone soil, the RAs of Proteobacteria and Acidobacteriota in healthy ones were decreased by 8.39% and 0.85%, respectively, while Firmicutes, Bacteroidota, Chloroflexi, and Actinobacteriota were increased by 113.76%, 8.71%, 4.89%, and 1.71%, respectively. Among the top 10 bacterial genera, *Sphingomonas*, *Vicinamibacteraceae, A4b*, *Gemmatimonadaceae*, and *Vicinamibacterales* were the main genera (Figure 5C). Compared with the diseased Chinese cabbage root-zone soil, the RAs of *Sphingomonas* and *Vicinamibacteraceae* in healthy ones were decreased by 3.51% and 1.48%, respectively, while *A4b*, *Vicinamibacterales,* and *Gemmatimonadaceae* were increased by 15.52%, 1.62%, and 1.35%, respectively. Similarly, in the fungal community, Ascomycota, Basidiomycota, Chytridiomycota, and Mortierellomycota were the main phyla (Figure 5B). Compared with the diseased Chinese cabbage root-zone soil, the RAs of Mortierellomycota and Ascomycota in healthy ones were significantly increased by 50.72% and 31.22%, respectively, while Chytridiomycota and Basidiomycota were significantly decreased by 23.58% and 21.80%, respectively. Among the top 10 fungal genera, *Podospora*, *Plectosphaerella*, *Agaricomycetes*, and *Mortierella* were the main phyla (Figure 5D). Compared with the diseased Chinese cabbage root-zone soil, the RAs of *Podospora* and *Mortierella* in healthy ones were significantly increased by 485.08% and 51.59%, respectively, while *Plectosphaerella* and *Agaricomycetes* were decreased by 86.22% and 22.57%, respectively. In other words, compared to the diseased Chinese cabbage root-zone soil, the microbial community composition of the healthy Chinese cabbage root-zone soil was reconstructed.

**Figure 6:**
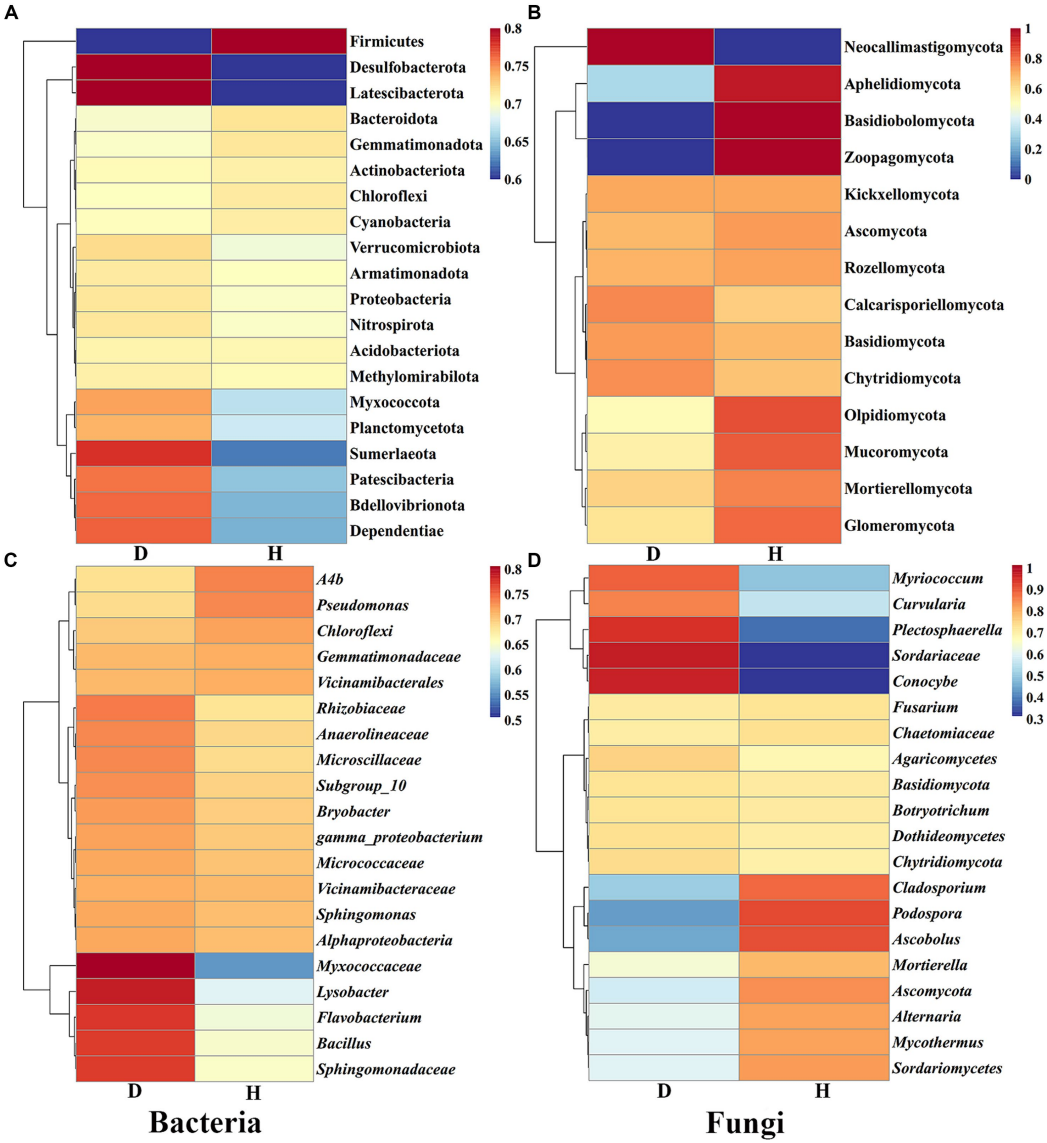
The heat maps show the abundance of the dominating bacterial **(A,C)** and fungal **(B,D)** communities at the phylum and genus levels, respectively.

Furthermore, heat maps provided a visual explanation for the variation in RAs composition (phylum and genus level) between the Chinese cabbage root-zone soil microbial community in healthy and sick circumstances (Figure 6). The root-zone soil of healthy Chinese cabbage exhibited a significant enrichment of the bacterial phylum Firmicutes, while the phyla Desulfobacterota, Latescibacterota, Sumerlaeota, Patescibacteria, Bdellovibrionota, and Dependentiae were notably decreased. Within the bacterial genus level, *Ab4*, *Pseudomonas*, *Chloroflexi*, *Gemmatimonadaceae,* and *Vicinamibacterales* showed higher abundances in the root-zone soil of healthy Chinese cabbage, whereas *Myxococcaceae*, *Lysobacter*, *Flavobacterium*, *Bacillus,* and *Sphingomonadaceae* were significantly reduced. Moving to the fungal community, the phyla Aphelidiomycota, Basidiobolomycota, and Zoopagomycota were more abundant in the root-zone soil of healthy Chinese cabbage, while Neocallimastigomycota exhibited a significant decrease in abundance. At the fungal genus level, the root-zone soil of healthy Chinese cabbage exhibited enrichment in *Cladosporium*, *Podospora*, *Ascobolus*, *Mortierella*, *Ascomycota*, *Alternaria*, *Mycothermus,* and *Sordariomycetes.* Conversely, there was a significant reduction in the presence of *Myriococcum*, *Curvularia*, *Plectosphaerella*, *Sordariaceae,* and *Conocybe.* The results demonstrated that soft rot disease has a discernible impact on the absence or presence of specific microbial taxa, and Chinese cabbage might keep themselves healthy through the alteration of root-zone soil microbial communities.

**Figure 7:**
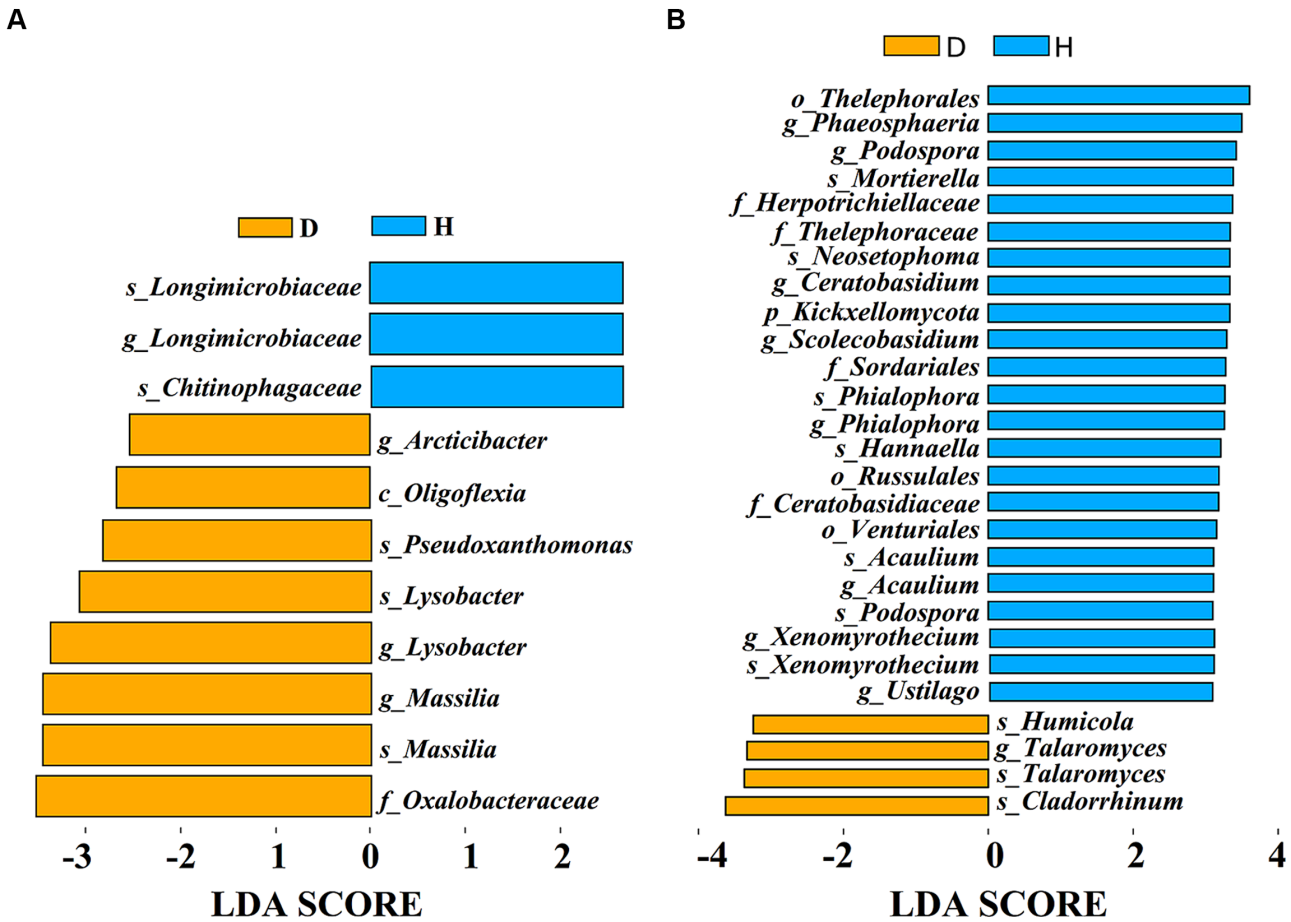
The effect size (LEfSe) of Liner discriminant analysis (LDA) on the bacterial **(A)** and fungal **(B)** taxa. Only fungal taxa with LDA values > 3 and bacterial taxa with LDA values > 2.5 (*p* < 0.05) are displayed.

To identify specific microbial biomarkers distinguishing between the Chinese cabbage root-zone soil microbial communities under diseased and healthy conditions, LEfSe analysis was conducted (Figure 7). The results revealed 11 bacterial biomarkers (LDA > 2.5) and 27 fungal biomarkers (LDA > 3) present in the root-zone soil of both diseased and healthy Chinese cabbage. In fact, two varieties of Longimicrobiaceae and Chitinophagaceae were found in higher concentrations in the root-zone soil of healthy Chinese cabbage compared to diseased Chinese cabbage, which included higher concentrations of *Arcticibacter*, Oligoflexia, Pseudoxathomonas, two varieties of *Lysobacter* and *Massilia*, and Oxalobacteraceae (Figure 7A). Meanwhile, the root-zone soil of healthy Chinese cabbage was enriched with Thelephorales, *Phaeosphaeria*, two types of *Podospora*, *Mortierella*, Herpotrichiellaceae, Thelephoraceae, *Neosetophoma*, *Ceratobasidium*, Kickxellomycota, *Scolecobasidium*, Sordariales, two types of *Phialophora*, *Hannaella*, Russulales, Ceratobasidiaceae, Venturiales, two types of *Acaulium*, two types of *Xenomyrothecium*, and *Ustilago*, while diseased Chinese cabbage was also enriched with *Humicola*, two types of *Talaromyces*, and *Cladorrhinum* (Figure 7B).

### 3.3 Isolation and identification of PGPB from diseased Chinese cabbage root-zone soil

**Figure 8:**
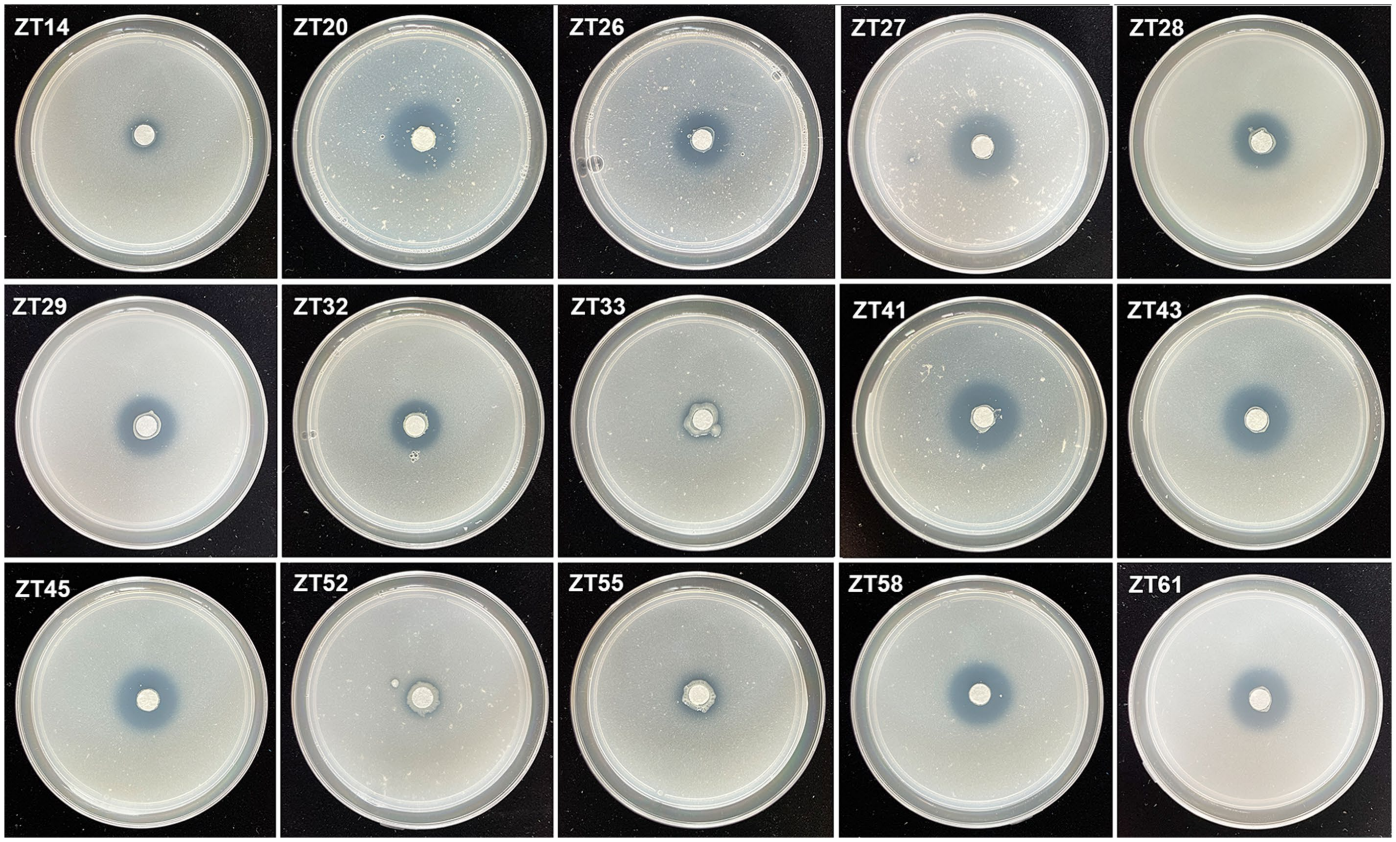
Phosphate solubilization assays of 15 strains on NBRIP media (showing the production of halo zone after 3 days of incubation).

**Figure 9:**
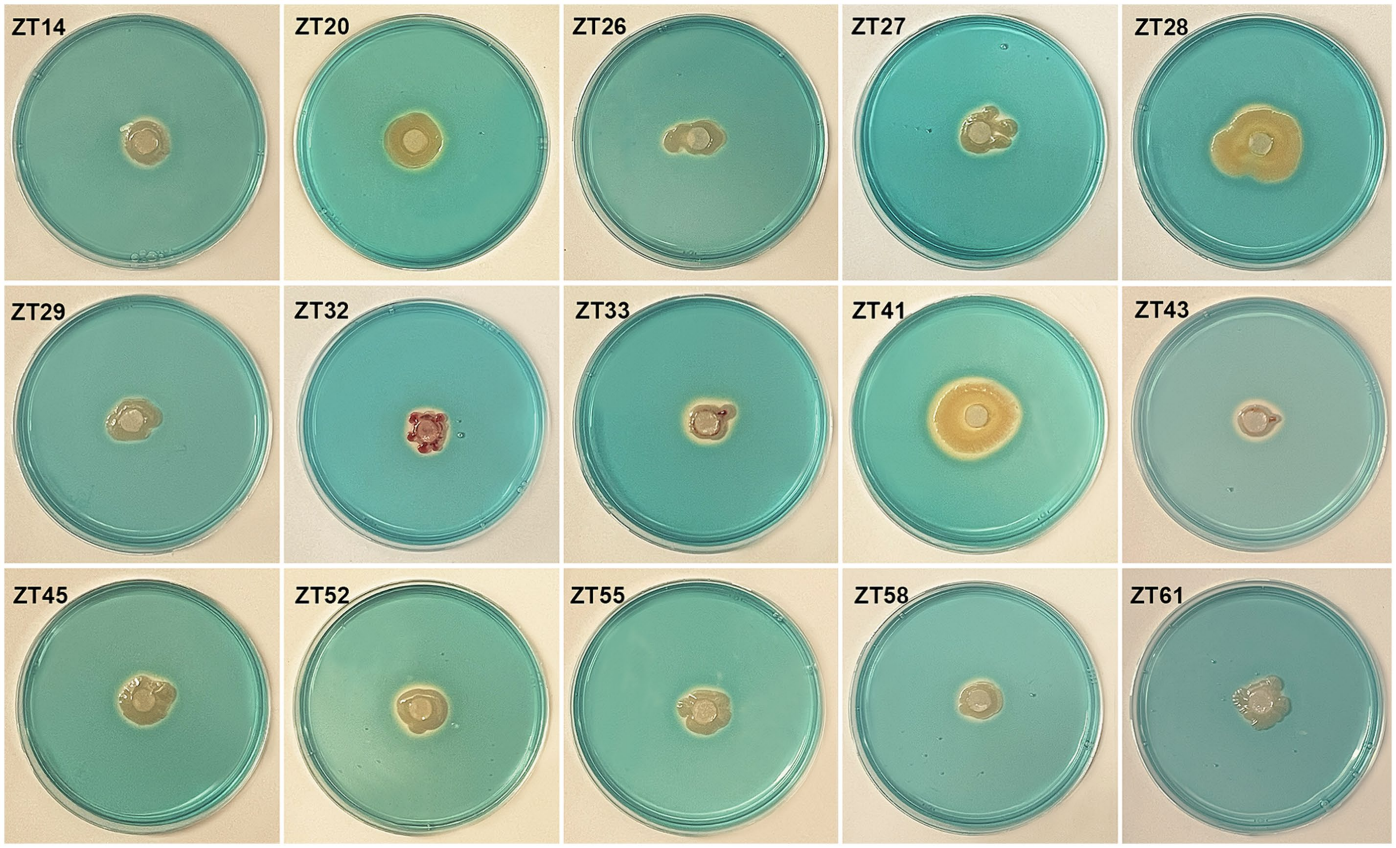
Evaluate the siderophore production of the 15 strains on CAS (Chrome Azurol S) media. The presence of an orange halo after 3 days of incubation indicated siderophore production.

To isolate plant-growth-promoting microbes, a total of 12 root-zone soil samples were collected from diseased Chinese cabbage. From these samples, a total of 588 bacterial strains were obtained. Further analysis was conducted using phosphate solubilization and siderophore production assays, which revealed that 15 strains exhibited both clear phosphate solubilizing zones and orange halos around their colonies after 3 days of incubation (Figures 8, 9; Table 1). The diameter of the phosphate solubilizing zones for each of the 15 strains was measured as follows: ZT41—18.81 mm, ZT43—18.64 mm, ZT58—18.26 mm, ZT45—18.09 mm, ZT27—18.06 mm, ZT29—17.19 mm, ZT61—16.66 mm, ZT26—15.65 mm, ZT28—14.67 mm, ZT20—14.35 mm, ZT33—11.85 mm, ZT55—11.28 mm, ZT32—11.06 mm, ZT52—10.61 mm, and ZT14—9.83 mm. Among the tested strains, ZT41 exhibited the largest phosphate solubilizing zone, which was not significantly different from ZT43, ZT58, ZT45, ZT27, and ZT29 but significantly larger than the other strains. Additionally, the diameters of the orange halos produced by all 15 strains were as follows: ZT28—14.99 mm, ZT41—14.57 mm, ZT26—14.38 mm, ZT20—11.52 mm, ZT55—9.98 mm, ZT45—9.65 mm, ZT27—9.40 mm, ZT52—9.10 mm, ZT43—8.97 mm, ZT33—8.94 mm, ZT61—8.78 mm, ZT58—8.66 mm, ZT29—8.45 mm, ZT14—8.42 mm, and ZT32—8.15 mm. ZT28 produced the largest orange halo, which was not significantly different from ZT41 and ZT26 but significantly larger than the other strains. These results indicate that the 15 strains have potential applications in the development of phosphate solubilization and siderophore production agents.

**Table 1:** The 15 isolated bacterial strains’ abilities in phosphate solubilization, siderophore generation, nitrogen fixation, and IAA production.

Strains	Phosphate solubilization ability (mm)	Siderophore production (mm)	IAA (μg/mL)		Nitrogen fixation
			0.1%	1.0%	
ZT14	9.83 ± 0.85 d	8.42 ± 0.68 d	37.41 ± 3.36 cd	63.95 ± 4.17 g	+
ZT20	14.35 ± 3.13 c	11.52 ± 0.76 b	30.46 ± 2.30 e	62.11 ± 4.31 g	−
ZT26	15.65 ± 0.91 bc	14.38 ± 2.38 a	57.26 ± 2.59 a	127.99 ± 8.76 c	−
ZT27	18.06 ± 1.07 a	9.40 ± 1.00 cd	37.33 ± 3.56 cd	51.99 ± 2.80 hi	+
ZT28	14.67 ± 0.83 c	14.99 ± 1.48 a	40.25 ± 1.88 bc	45.97 ± 1.93 ij	+
ZT29	17.19 ± 5.63 ab	8.45 ± 0.99 d	28.21 ± 1.27 e	57.90 ± 3.40 gh	++
ZT32	11.06 ± 0.59 d	8.15 ± 0.37 d	28.46 ± 3.51 e	44.32 ± 1.54 ij	++
ZT33	11.85 ± 0.29 d	8.94 ± 0.34 cd	31.86 ± 3.29 de	64.38 ± 2.72 g	++
ZT41	18.81 ± 2.51 a	14.57 ± 1.27 a	31.05 ± 2.05 e	15.38 ± 3.91 k	−
ZT43	18.64 ± 0.56 a	8.97 ± 1.39 cd	37.42 ± 2.66 cd	73.55 ± 4.67 f	+
ZT45	18.09 ± 1.05 a	9.65 ± 1.06 cd	36.61 ± 3.67 cd	86.29 ± 4.11 de	−
ZT52	10.61 ± 0.48 d	9.10 ± 0.90 cd	52.34 ± 3.73 a	233.07 ± 6.63 a	+
ZT55	11.28 ± 0.90 d	9.98 ± 0.67 c	45.35 ± 3.97 b	143.33 ± 4.70 b	++
ZT58	18.26 ± 0.68 a	8.66 ± 1.14 cd	51.85 ± 4.84 a	83.14 ± 2.49 e	+
ZT61	16.66 ± 0.67 abc	8.78 ± 0.30 cd	38.68 ± 5.01 c	92.60 ± 3.44 d	−

Values that are separated by distinct lowercase letters within the same column indicate a significant difference at *p* < 0.05. −, none; +, mild; ++, strong.

**Figure 10:**
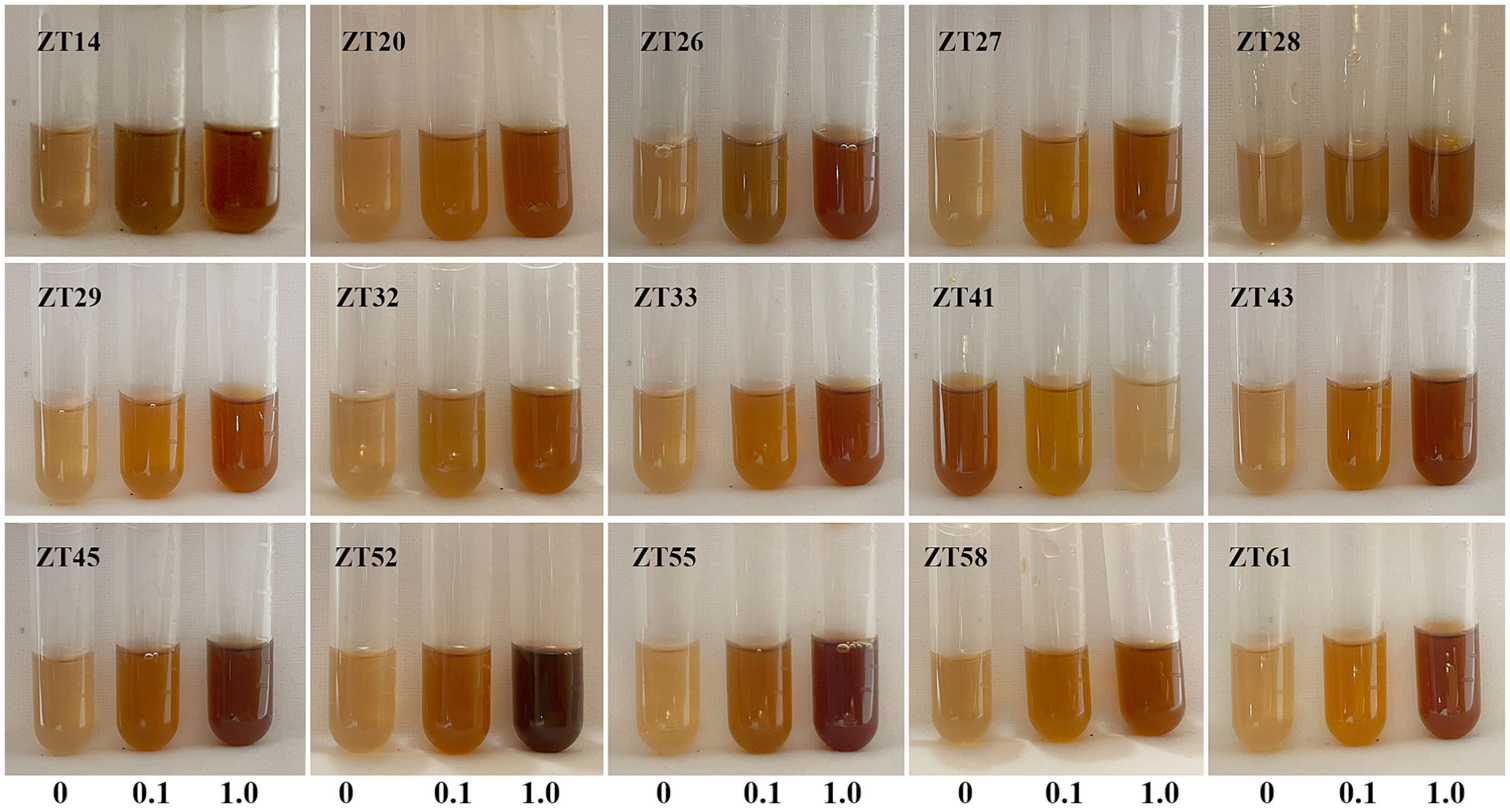
Evaluate the IAA production of the 15 strains on LB medium supplemented with either 0.1% or 1.0% tryptophan. The development of a pink-red color after incubation indicated IAA production.

**Figure 11:**
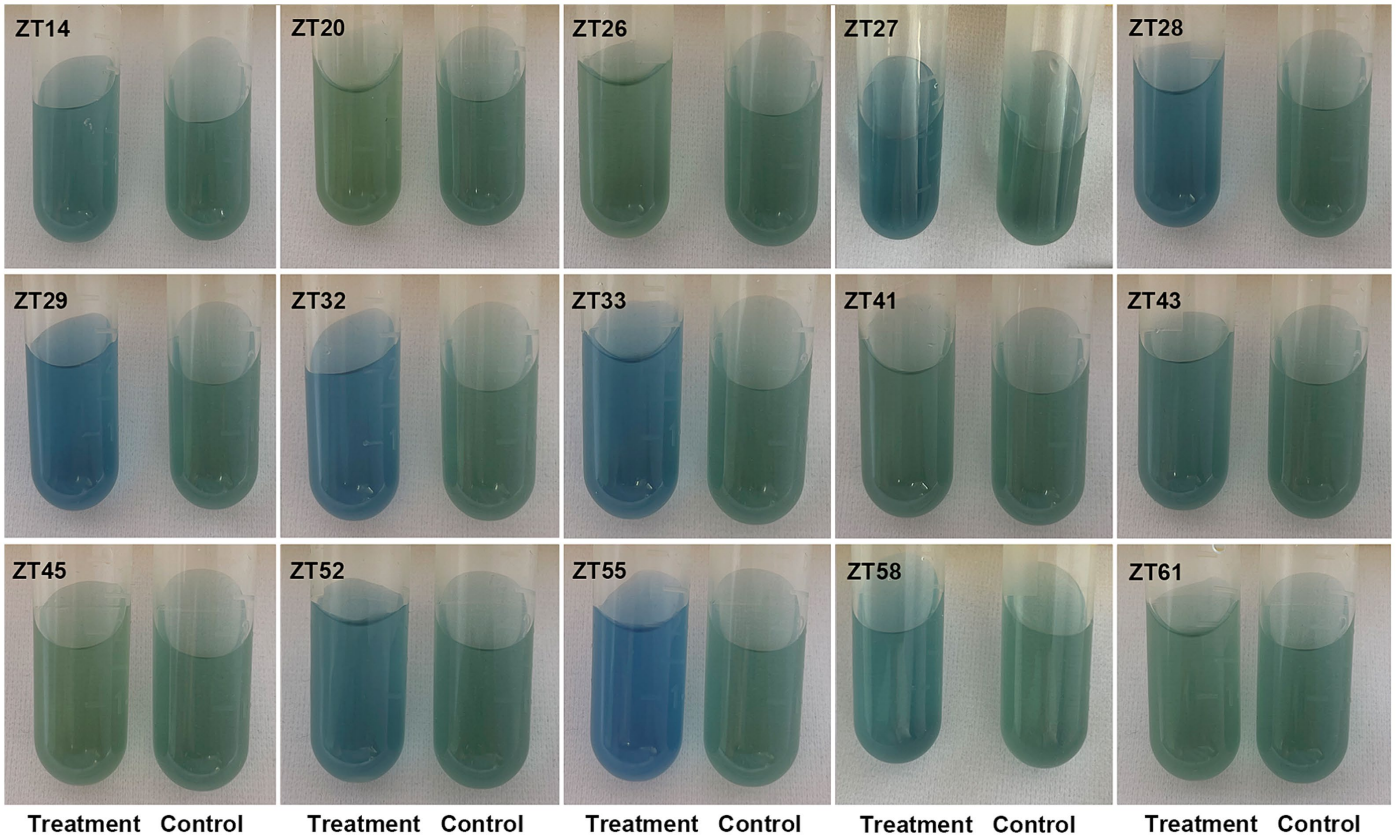
Evaluate the nitrogen fixation of the 15 strains on the Nfb medium. After incubation, the color of the test tubes changed from green to blue, indicating nitrogen fixation.

In addition to phosphate solubilization and siderophore production, the 15 strains were also evaluated for their IAA production and nitrogen fixation ability (Figures 10, 11; Table 1). The results showed variations in IAA production among the 15 strains when grown in LB supplemented with 0.1% or 1.0% tryptophan. Specifically, the amounts of IAA produced by the 15 strains were as follows at 0.1% tryptophan: ZT26—57.26 μg/mL, ZT52—52.34 μg/mL, ZT58—51.85 μg/mL, ZT55—45.35 μg/mL, ZT28—40.25 μg/mL, ZT61—38.68 μg/mL, ZT43—37.42 μg/mL, ZT14—37.41 μg/mL, ZT27—37.33 μg/mL, ZT45—36.61 μg/mL, ZT33—31.86 μg/mL, ZT41—31.05 μg/mL, ZT20—30.46 μg/mL, ZT32—28.46 μg/mL, and ZT29—28.21 μg/mL. At 1.0% tryptophan, the amounts of IAA produced by the 15 strains were as follows: ZT52—233.07 μg/mL, ZT55—143.33 μg/mL, ZT26—127.99 μg/mL, ZT61—92.60 μg/mL, ZT45—86.29 μg/mL, ZT58—83.14 μg/mL, ZT43—73.55 μg/mL, ZT33—64.38 μg/mL, ZT14—63.95 μg/mL, ZT20—62.11 μg/mL, ZT29—57.90 μg/mL, ZT27—51.99 μg/mL, ZT28—45.97 μg/mL, ZT32—44.32 μg/mL, and ZT41—15.38 μg/mL. These results indicate that the production of IAA is influenced by both the strains and the concentrations of tryptophan in the LB medium. Furthermore, except for ZT20, ZT26, ZT41, ZT45, and ZT61, the other 10 strains were capable of changing the color of NfB media from green to blue after 2 days of incubation. This color change indicates that these 10 strains have the ability to fix atmospheric nitrogen.

Through the examination of colony morphology and conducting BLAST searches of 16S rRNA gene sequences against the NCBI GenBank database, all 15 strains were subjected to further identification. The results revealed that strains ZT20 and ZT33 displayed 100.00% sequence similarity and were identified as *Enterobacter cloacae*. Similarly, strains ZT26 and ZT45 exhibited 99.57 and 99.50% sequence similarity, respectively, and were identified as *Klebsiella aerogenes*. Strains ZT28, ZT43, and ZT58 shared 99.79% sequence similarity and were identified as *Klebsiella pasteurii*. Strains ZT27 and ZT41 showed 98.93% and 99.01% sequence similarity, respectively, and were identified as *Myroides odoratimimus*. Furthermore, strains ZT14, ZT29, ZT32, ZT52, ZT55, and ZT61 were identified as *Citrobacter freundii*, *Enterobacter hormaechei*, *Pseudomonas putida*, *Bacillus paramycoides*, *Peribacillus asahii*, and *Pantoea coffeiphila*, respectively, with sequence similarities of 100.00%, 99.93%, 100.00%, 99.46%, 99.93%, and 99.09%, as indicated (Table 2).

**Table 2:** Identification of 15 strains based on analysis of 16S rRNA sequence.

Strains	Bacterial identify	Sequence similarity (%)	Accession number
ZT14	*Citrobacter freundii*	100.00	PP429531
ZT20	*Enterobacter cloacae*	100.00	PP429532
ZT26	*Klebsiella aerogenes*	99.57	PP429533
ZT27	*Myroides odoratimimus*	98.93	PP429534
ZT28	*Klebsiella pasteurii*	99.79	PP429535
ZT29	*Enterobacter hormaechei*	99.93	PP429536
ZT32	*Pseudomonas putida*	100.00	PP429537
ZT33	*Enterobacter cloacae*	100.00	PP429538
ZT41	*Myroides odoratimimus*	99.01	PP542492
ZT43	*Klebsiella pasteurii*	99.79	PP429540
ZT45	*Klebsiella aerogenes*	99.50	PP429541
ZT52	*Bacillus paramycoides*	99.46	PP429542
ZT55	*Peribacillus asahii*	99.93	PP429543
ZT58	*Klebsiella pasteurii*	99.79	PP429544
ZT61	*Pantoea coffeiphila*	99.09	PP429545

### 3.4 Effect of PGPB on Chinese cabbage growth under soft rot stress

**Figure 12:**
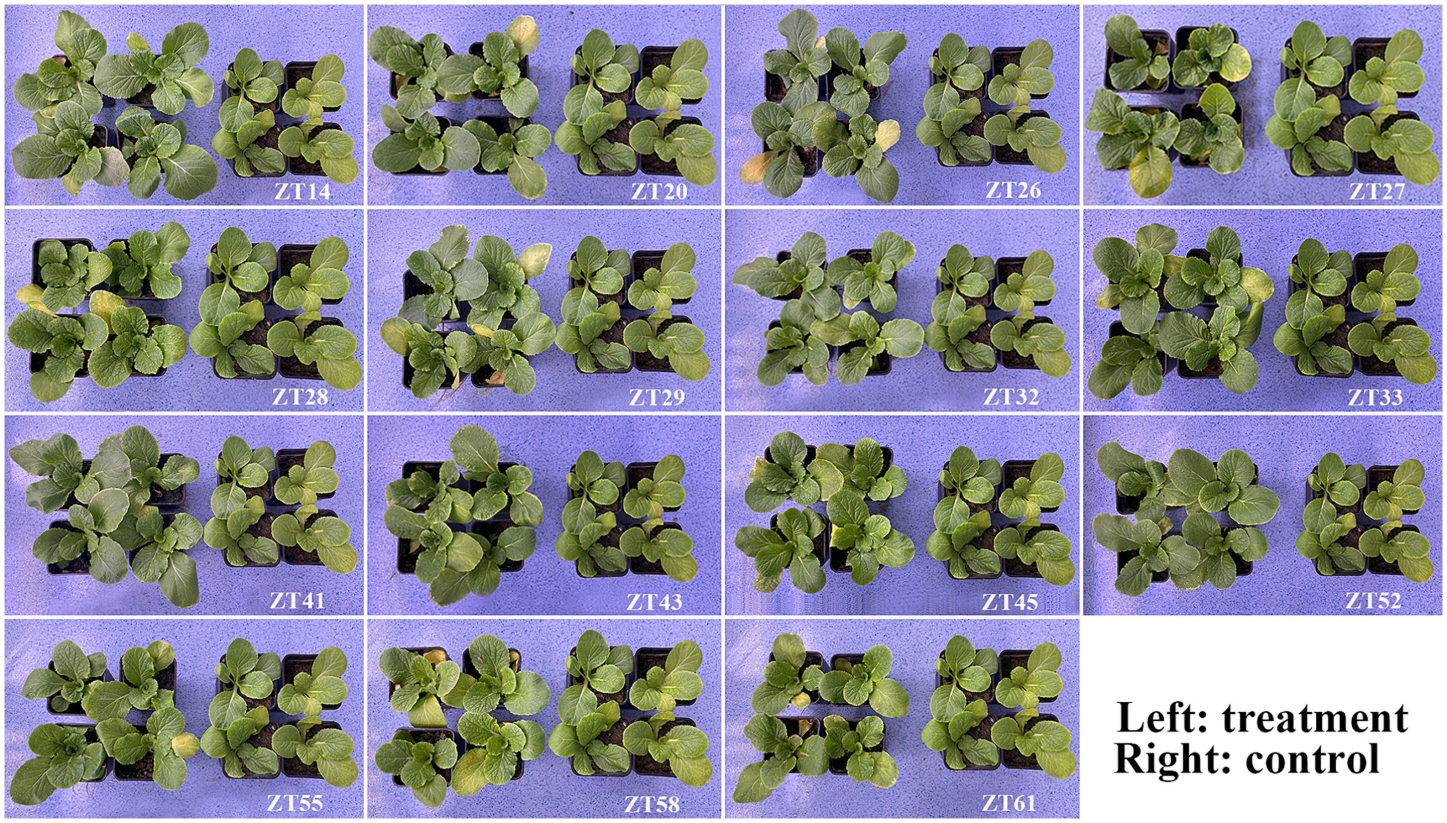
Effect of 15 bacterial strains on Chinese cabbage growth in soil under soft rot stress 28 days after inoculation.

**Figure 13:**
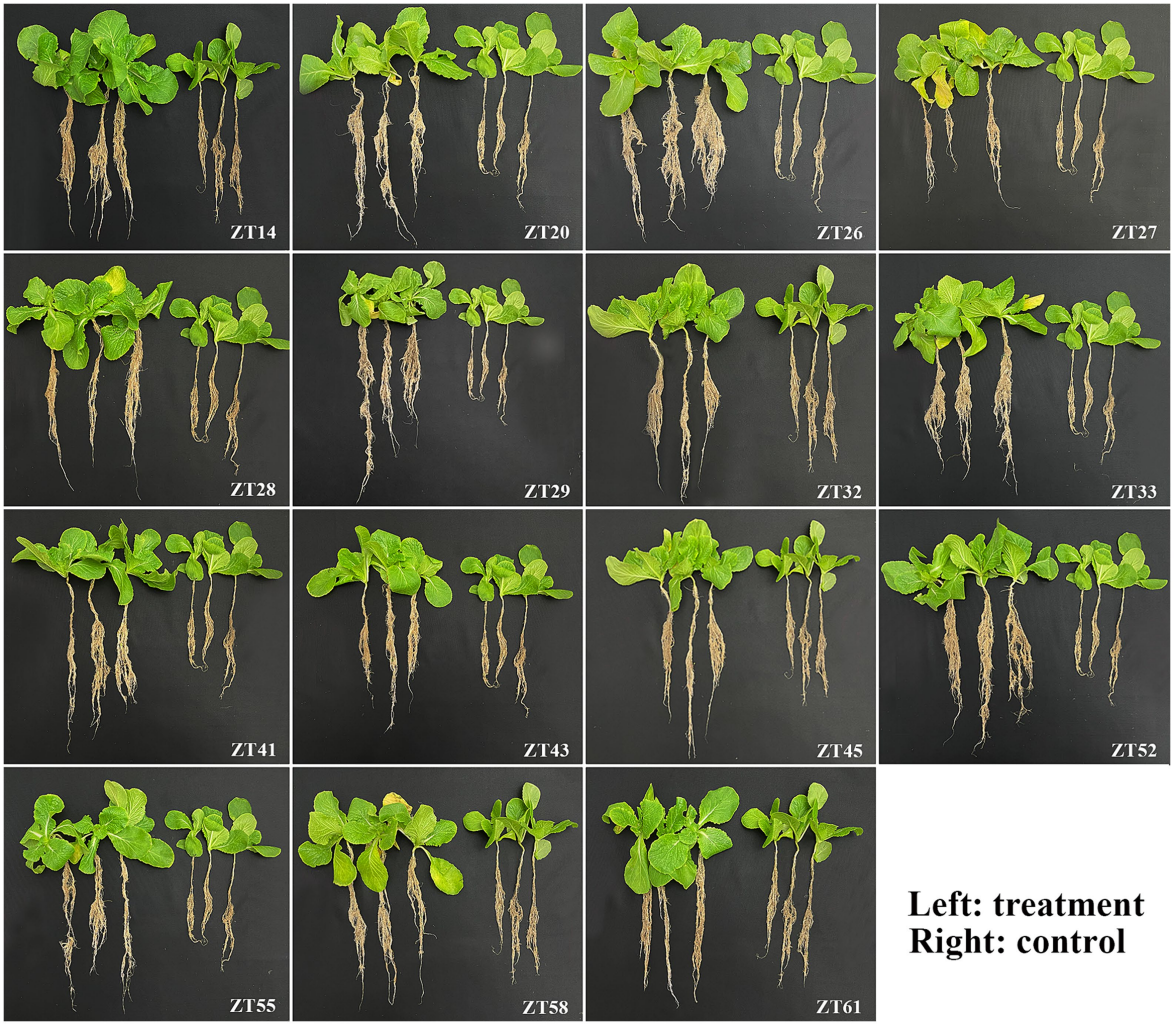
Effect of 15 bacterial strains on Chinese cabbage root growth in soil under soft rot stress 28 d after inoculation.

To evaluate the influence of 15 bacterial strains on the growth of Chinese cabbage under soft rot stress, plant growth promotion (PGP) tests were conducted in a greenhouse, as depicted in Figures 12, 13. The outcomes indicated that all 15 strains exhibited varying degrees of significant promotion of Chinese cabbage growth. Through phenotypic observations, it was evident that all strains, except for ZT27, which resulted in a 5.71% reduction, caused a noticeable increase (ranging from 6.29% to 30.29%) in leaf number and leaf size compared to the control group. Furthermore, the measured data demonstrated that all 15 strains had a significant impact on root development and biomass aggregation in Chinese cabbage when compared to the control, as detailed in Table 3. Among the tested strains, ZT14 exhibited a significant impact on the growth of Chinese cabbage seedlings. It resulted in a remarkable 214.88% increase in fresh weight and a 165.08% increase in dry weight for seedlings. Additionally, ZT14 led to a 13.82% increase in root length, as well as substantial increases of 461.41% and 117.64% in fresh and dry weight of roots, respectively, compared to the control. Similarly, ZT20 demonstrated significant growth-promoting effects on Chinese cabbage seedlings, with a 92.68% increase in fresh weight and a 56.02% increase in seedling dry. The strain also induced a 16.96% increase in root length, accompanied by significant increases of 209.02% and 10.97% in fresh and dry weight of roots, respectively, compared to the control. ZT26 also had a notable impact on Chinese cabbage growth, resulting in a 127.21% increase in fresh weight and an 84.82% increase in dry weight of seedlings. Moreover, ZT26 stimulated a 9.65% increase in root length, along with substantial increases of 516.47% and 179.88% in fresh and dry weight of roots, respectively, compared to the control. In contrast, ZT27 exhibited differing effects on Chinese cabbage growth. It led to a 91.58% increase in fresh weight and a 41.00% increase in dry weight of seedlings. However, ZT27 caused a decrease of 19.68% in root length and a decrease of 16.86% in dry weight of roots compared to the control. Interestingly, ZT27 resulted in a 19.41% increase in the fresh weight of roots compared to the control. ZT28 demonstrated a considerable impact, resulting in a 163.62% increase in fresh weight and a 140.50% increase in dry weight of seedlings. Additionally, ZT28 led to an 11.03% increase in root length, along with significant increases of 216.71% and 43.06% in fresh and dry weight of roots, respectively, compared to the control. Similarly, ZT29 exhibited substantial growth-promoting effects, with a 121.83% increase in fresh weight and a 97.57% increase in dry weight of seedlings. ZT29 also contributed to a 34.84% increase in root length, along with significant increases of 274.59% and 108.45% in fresh and dry weight of roots, respectively, compared to the control. ZT32 had a notable impact on Chinese cabbage growth, resulting in a 129.59% increase in fresh weight and a 91.34% increase in dry weight of seedlings. Moreover, ZT32 stimulated a 13.11% increase in root length, accompanied by significant increases of 397.88% and 136.78% in fresh and dry weight of roots, respectively, compared to the control. ZT33 exhibited remarkable growth-promoting effects on Chinese cabbage, leading to an 184.69% increase in fresh weight and a 154.65% increase in dry weight of seedlings. Furthermore, ZT33 resulted in a 27.17% increase in root length, along with significant increases of 651.06% and 272.33% in fresh and dry weight of roots, respectively, compared to the control. ZT41 also demonstrated significant growth-promoting effects, with a 151.94% increase in fresh weight and a 94.81% increase in dry weight of seedlings. The strain induced a 19.47% increase in root length, along with significant increases of 396.00% and 81.32% in fresh and dry weight of roots, respectively, compared to the control. ZT43 had a positive impact on Chinese cabbage growth, resulting in a 107.42% increase in fresh weight and a 34.91% increase in dry weight of seedlings. Additionally, ZT43 stimulated a 7.04% increase in root length, along with significant increases of 194.59% and 22.98% in fresh and dry weight of roots, respectively, compared to the control. ZT45 exhibited significant growth-promoting effects, with a 131.98% increase in fresh weight and a 100.30% increase in dry weight of seedlings. Furthermore, ZT45 led to a 14.89% increase in root length, accompanied by significant increases of 365.41% and 76.92% in fresh and dry weight of roots, respectively, compared to the control. ZT52 contributed to Chinese cabbage growth, resulting in a 141.51% increase in fresh weight and a 67.16% increase in dry weight of seedlings. The strain also induced a 33.70% increase in root length, along with significant increases of 341.18% and 91.02% in fresh and dry weight of roots, respectively, compared to the control. ZT55 exhibited growth-promoting effects, with an 88.06% increase in fresh weight and a 52.62% increase in dry weight of seedlings. Moreover, ZT55 stimulated a 13.37% increase in root length, along with significant increases of 187.53% and 21.86% in fresh and dry weight of roots, respectively, compared to the control. ZT58 demonstrated significant growth-promoting effects, resulting in a 137.44% increase in fresh weight and a 103.66% increase in dry weight of seedlings. Additionally, ZT58 led to a 12.19% increase in root length, accompanied by significant increases of 370.59% and 52.25% in fresh and dry weight of roots, respectively, compared to the control. ZT61 had a moderate impact on Chinese cabbage growth, resulting in a 65.29% increase in fresh weight and a 33.95% increase in dry weight of seedlings. Furthermore, ZT61 stimulated a 10.09% increase in root length, along with significant increases of 238.82% and 26.57% in fresh and dry weight of roots, respectively, compared to the control. These results underscore the good effects of different bacterial strains (ZT14, ZT33, ZT41, ZT52, ZT58, ZT45, and ZT32) on the growth parameters of Chinese cabbage seedlings under soft rot stress conditions, highlighting their potential for promoting plant growth and development.

**Table 3:** Effects of 15 bacterial strains on growth of Chinese cabbage.

Treatments	LN	GPE%	SFW (g)	GPE%	RFW (g)	GPE%
ZT14	7.60 ± 0.49	30.29 a	7.38 ± 0.75	214.88 a	2.39 ± 0.21	461.41 b
ZT20	5.83 ± 0.75	0.00 cd	4.52 ± 0.77	92.68 fgh	1.31 ± 0.14	209.02 de
ZT26	6.60 ± 0.49	13.14 b	5.33 ± 0.57	127.21 de	2.62 ± 0.29	516.47 b
ZT27	5.50 ± 0.45	−5.71 d	4.49 ± 0.52	91.58 fgh	0.51 ± 0.06	19.41 f
ZT28	6.80 ± 0.40	16.57 b	6.18 ± 0.68	163.62 bc	1.35 ± 0.21	216.71 de
ZT29	6.40 ± 0.49	9.71 bc	5.20 ± 0.24	121.83 def	1.59 ± 0.28	274.59 d
ZT32	6.80 ± 0.75	16.57 b	5.38 ± 0.56	129.59 de	2.12 ± 0.14	397.88 c
ZT33	6.60 ± 0.49	13.14 b	6.68 ± 0.38	184.69 b	3.19 ± 0.30	651.06 a
ZT41	6.80 ± 0.40	16.57 b	5.91 ± 0.80	151.94 cd	2.11 ± 0.22	396.00 c
ZT43	6.20 ± 0.40	6.29 bc	4.86 ± 0.31	107.42 efg	1.25 ± 0.15	194.59 e
ZT45	6.80 ± 0.40	16.57 b	5.44 ± 0.74	131.98 cde	1.98 ± 0.22	365.41 c
ZT52	6.50 ± 0.55	11.43 b	5.66 ± 0.67	141.51 cd	1.88 ± 0.16	341.18 c
ZT55	6.60 ± 0.49	13.14 b	4.41 ± 0.59	88.06 gh	1.22 ± 0.15	187.53 e
ZT58	6.80 ± 0.40	16.57 b	5.57 ± 0.43	137.44 cde	2.00 ± 0.33	370.59 c
ZT61	6.20 ± 0.40	6.29 bc	3.88 ± 0.28	65.29 h	1.44 ± 0.25	238.82 de
Control	5.83 ± 0.75	–	2.35 ± 0.64	–	0.43 ± 0.07	**–**
**Treatments**	**RL (mm)**	**GPE%**	**SDW (g)**	**GPE%**	**RDW (g)**	**GPE%**
ZT14	264.93 ± 11.13	13.82 def	0.92 ± 0.07	165.08 a	0.22 ± 0.02	117.64 cd
ZT20	272.23 ± 12.86	16.96 cd	0.54 ± 0.06	56.02 ef	0.11 ± 0.03	10.97 hi
ZT26	255.22 ± 8.03	9.65 fg	0.64 ± 0.04	84.82 cd	0.29 ± 0.05	179.88 b
ZT27	186.95 ± 7.08	−19.68 h	0.49 ± 0.02	41.00 f	0.09 ± 0.01	−16.86 j
ZT28	258.42 ± 8.70	11.03 efg	0.84 ± 0.11	140.50 b	0.15 ± 0.03	43.06 fgh
ZT29	313.85 ± 8.32	34.84 a	0.69 ± 0.05	97.57 c	0.22 ± 0.02	108.45 cde
ZT32	263.28 ± 9.31	13.11 def	0.67 ± 0.06	91.34 c	0.24 ± 0.03	136.78 c
ZT33	296.00 ± 4.86	27.17 b	0.89 ± 0.09	154.65 ab	0.38 ± 0.07	272.33 a
ZT41	278.07 ± 8.42	19.47 c	0.68 ± 0.07	94.81 c	0.19 ± 0.02	81.32 def
ZT43	249.15 ± 6.75	7.04 g	0.47 ± 0.03	34.91 f	0.13 ± 0.02	22.98 gh
ZT45	267.42 ± 7.20	14.89 cde	0.70 ± 0.07	100.30 c	0.18 ± 0.03	76.92 ef
ZT52	311.19 ± 11.65	33.70 a	0.58 ± 0.07	67.16 de	0.20 ± 0.02	91.02 de
ZT55	263.87 ± 11.23	13.37 def	0.53 ± 0.04	52.62 ef	0.13 ± 0.02	21.86 gh
ZT58	261.12 ± 7.27	12.19 def	0.71 ± 0.07	103.66 c	0.16 ± 0.03	52.25 fg
ZT61	256.24 ± 7.86	10.09 efg	0.47 ± 0.06	33.95 f	0.13 ± 0.03	26.57 gh
Control	232.76 ± 6.64	–	0.35 ± 0.03	–	0.10 ± 0.02	–

LN, leaf number; RL, root length; SFW, seedling fresh weight; SDW, seedling dry weight; RFW, root fresh weight; RDW, root dry weight; GPE, growth promotion efficacy. Means are averages ± SD. Different lowercase letters within the same columns reveal the significance among different treatments (*p* < 0.05).

## 4 Discussion

Bacterial soft rot, turnip mosaic virus, and downy mildew constitute the three major diseases of Chinese cabbage all over the world (Yang, 1988). Among these, bacterial soft rot poses a significant threat to Chinese cabbage crops, resulting in yield losses ranging from 20% to 80% in the field and causing substantial economic losses in China each year (Guo et al., 2019). *Pectobacterium* spp. is considered one of the most destructive plant pathogens globally and is frequently identified as the causal agent of bacterial soft rot in Chinese cabbage (Davidsson et al., 2013). In addition to *P. carotovorum* subsp. *carotovorum* (*Pcc*), *P. carotovorum* subsp. *brasiliense* (*Pcb*), *P. carotovorum* subsp. *odoriferum* (*Pco*), and *P. polaris*, they can also cause bacterial soft rot. Moreover, other bacteria such as *Pseudomonas* sp., *Xanthomonas* spp., and *Pantoea agglomerans* have also been reported as causal agents of bacterial soft rot (Guo et al., 2019; Lee et al., 2019; Chen et al., 2021). To address the significant losses caused by bacterial soft rot, extensive research has been conducted. Various molecular techniques have been employed for the identification and detection of soft-rot bacteria. For example, 16S rRNA gene sequencing can identify soft rot bacteria at the genus or species level, while phylogenetic analysis based on the *pmr*A gene serves as a rapid and efficient tool for subspecies identification. Additionally, specific PCR assays using primers such as Y1/Y2 (genus-specific), PhF/PhR (species-specific), and srlE-qF1/srlE-qR1 (subspecies-specific) have been developed for rapid identification of soft rot pathogens (Darrasse et al., 1994; Naum et al., 2008; De Boer et al., 2012; Kettani-Halabi et al., 2013; Li et al., 2020). In efforts to mitigate the severity of soft rot, the introduction of specific genes has been explored. For instance, the introduction of the AtWRKY75 gene into Chinese cabbage has been shown to reduce susceptibility to *Pcc* infection (Choi et al., 2016). Traditional antimicrobial agents, such as copper-based bactericides and antibiotics, have been used for controlling soft rot-bacteria. However, their use has been limited due to microbial resistance, accumulation on plants, and potential hazards to human health (Abd-EI-Khair et al., 2021; Vu et al., 2024). In recent years, there has been growing interest in biological control methods to manage bacterial soft rot. These approaches involve the application of beneficial microorganisms such as *Trichoderma* spp., *Bacillus* spp., *Pseudomonas* spp., *Lactobacillus* spp., and bacteriophages (Tsuda et al., 2016; Abd-EI-Khair et al., 2021; Vu et al., 2024). These biological control agents can help combat soft rot by competing with the pathogens, producing antimicrobial compounds, or inducing plant defenses. Microbes play a crucial role in agroecosystems by influencing microbial communities, maintaining soil fertility, promoting stress tolerance, and enhancing plant growth (Harmann and Six, 2023). In our study conducted in Hangzhou, China, we focused on investigating the pathogen responsible for causing soft rot in Chinese cabbage. The isolated pathogen was identified, and we further examined the variations in microbial communities present in the root-zone soil of both healthy and diseased Chinese cabbage affected by bacterial soft rot. Additionally, we carried out a systematic isolation and study of plant-growth-promoting bacteria.

### 4.1 The pathogen of soft rot on Chinese cabbage

In our study conducted in Hangzhou, China, we utilized 16S rRNA and *pmr*A gene sequence analysis to identify the pathogen responsible for soft rot on Chinese cabbage. The analysis revealed that the pathogen causing soft rot in this region is identified as *P. brasiliense*. Previous research has shown that the majority of soft rot cases in Chinese cabbage are typically caused by *P. carotovorum* subsp. *carotovorum* (*Pcc*), *P. carotovorum* subsp. *brasiliense* (*Pcb*), and *P. carotovorum* subsp. *odoriferum* (*Pco*; Li et al., 2020). Therefore, *Pcb* has been elevated to the species *P. brasiliense* by phylogeny analysis with other completely sequenced *Pectobacterium* species (Portier et al., 2019; Oulghazi et al., 2021). Recent reports have consistently highlighted *P. brasiliense* as a highly aggressive pathogen, causing significant losses in various plant species from 10 different families, including potato, cucumber, and tomato (De Werra et al., 2015; Meng et al., 2017; Voronina et al., 2019; Oulghazi et al., 2021). Consequently, *P. brasiliense* is considered to be a particularly virulent pathogen, causing more instances of soft rot disease compared to other *Pectobacterium* species (Van der Wolf et al., 2017).

### 4.2 Differences between root-zone microbiomes

In our study, we employed high-throughput sequencing to investigate the bacterial and fungal communities present in the root-zone soil of both healthy and diseased Chinese cabbage. Our results revealed that the bacterial community richness was higher in the diseased Chinese cabbage root-zone soil compared to the healthy soil, whereas the fungal community richness was higher in the healthy Chinese cabbage root-zone soil than in the diseased soil. Interestingly, the *α*-diversity of soil microbes in both the healthy and diseased Chinese cabbage root-zone soil did not exhibit a significant difference. These findings align with previous studies that have observed similar changes in bacterial diversity in the soil of *Lycium barbarum* and fungal diversity in the soil of *Panax notoginseng* caused by root rot (Wu Z. et al., 2015; Jia et al., 2023). PCA analysis also showed that there was some difference in the bacterial and fungal community composition of each soil sample from the healthy and diseased Chinese cabbage, which was also in line with Jia et al. (2023). Results also showed that the proportion of the top 10 bacteria and fungi in the root-zone soil of the healthy and diseased Chinese cabbage was quite different at the phylum and genus level. Indeed, compared with the diseased plants, the predominant phyla of Proteobacteria (−8.39%), Chloroflexi (4.89%), Bacteroidota (8.71%), Firmicutes (113.76%), Chytridiomycota (−23.58%), Basidiomycota (−21.80%), Ascomycota (31.22%), and Mortierellomycota (50.72%), and the predominant genera of *Sphingomonas* (−3.51%), *A4b* (15.52%), *Plectosphaerella* (−86.22%), *Agaricomycetes* (−22.57%), *Mortierella* (51.59%), and *Podospora* (485.08%), were significantly changed in the healthy ones. It indicated that the microbial community of the healthy Chinese cabbage was reconstructed, and more attention should be paid to the above microbes. Proteobacteria are very important to carbon, nitrogen, and cycling (Kersters et al., 2006). Chloroflexi can encode carbon monoxide dehydrogenases and hydrogenases, thereby utilizing carbon monoxide and molecular hydrogen to sustain growth and persistence (Islam et al., 2019). Bacteroidota are important and dominant carbohydrate degraders in the soil, and they have been considered to be sensitive biological indicators of the soil (Wolińska et al., 2017; Larsbrink and McKee, 2020). Enrichment of protective microbes in the rhizosphere can facilitate disease suppression; for example, the abundance of Firmicutes and Actinobacteria is lower in tomato bacterial wilt rhizosphere soil than in healthy ones (Lee et al., 2020). Some Chytridiomycota have resistant structures that enable survival under stressful environments such as drying and heat (Gleason et al., 2004). Ascomycetes and Basidiomycetes are very efficient in nutrient uptake, soil aggregation, and plant residue decomposition (Manici et al., 2024). Mortierellomycota can secrete and synthesize oxalic acid to dissolve mineral phosphorus into available phosphorus (Gai et al., 2021). *Sphingomonas* are important biocatalysts for soil bioremediation due to their unique catabolic capabilities for pollutants and their ability to produce beneficial phytohormones to improve plant growth under stressful conditions (Leys et al., 2004; Asaf et al., 2020). Most *Plectosphaerella* are pathogens causing large losses of pumpkin, melon, zucchini, pepper, tomato, bamboo, and asparagus (Yang et al., 2021). *Agaricomycetes* play a pivotal role in cycling nutrients in forest soils and bioremediating contaminated soil (Kersten and Culen, 2013; Mohammadi-Sichani et al., 2017). *Mortierella* have various characteristics supporting the defense mechanisms in plants, promoting plant growth, and reshaping the soil microbiological community (Ozimek and Hanaka, 2020). *Podospora* is usually abundant in healthy soil and can decay recalcitrant lignocelluloses (Couturier et al., 2016). Overall, beneficial microbes in the root zone of Chinese cabbage may provide the first line of defense against soft rot disease through competition or antagonism, increase biological activity under soft rot stress, improve soil structure, and promote plant growth by fixing atmospheric nitrogen and solubilizing phosphorus.

### 4.3 Evaluation of PGPB on Chinese cabbage growth

In screening for bacterial strains isolated from Chinese cabbage using selective media assays, a total of 15 bacterial strains were particularly active. The PGP tests also showed that the 15 strains could significantly promote plant growth at different levels in the greenhouse. Among them, the growth-promoting effect was particularly obvious in ZT14 (*Citrobacter freundii*), ZT33 (*Enterobacter cloacae*), ZT41 (*Myroides odoratimimus*), ZT52 (*Bacillus paramycoides*), ZT58 (*Klebsiella pasteurii*), ZT45 (*Klebsiella aerogenes*), and ZT32 (*Pseudomonas putida*). As we know, agriculturists have immensely applied commercial chemical fertilizers and pesticides to increase crop yield and lessen negative effects (such as several diseases due to pathogens and pests) in the past. However, long-term heavy use has brought serious ecological problems and pest resistance. Nowadays, more and more attention has been paid to beneficial microbes (the core of biofertilizers and biopesticides) in view of their ability to support plants and prevent pathogens in an environmentally friendly way (Ortiz and Sansinenea, 2022). Fadiji et al. (2023) showed that *C. freundii* possesses multiple PGP traits (such as solubilization of phosphate, production of ammonia and siderophores, and indole-3-acetic acid) and can be used as a biofertilizer in agriculture. Mehnaz et al. (2010) used *E. cloacae* and *P. putida* as inoculums for corn in a greenhouse, and growth promotion on root and shoot weight was observed. *Myroides odoratimimus* can improve plant growth (such as mung bean and wheat) by increasing chemical content and plant growth hormone, as well as restrain important pests and pathogen development in agriculture (Zhang et al., 2022). *Bacillus* spp. are well-known for their capacity to counter plant pathogens and enhance plant development by producing hydrogen cyanide and catalase (Mghazli et al., 2022). For example, *B. paramycoides* can effectively enhance soil health by inhibiting the production of harmful soil metabolites and improving soybean tolerance to root rot disease (Yang et al., 2023). *Klebsiella* sp. can improve the growth and yield of maize, and *Klebsiella* MK2R2 can enhance tolerance to salinity and promote oat seedling growth (Sapre et al., 2017; Mowafy et al., 2021). *K. aerogenes* can enhance *Bacopa monnieri* growth through nitrogen fixation (Shukla et al., 2022). Overall, the PGPMs-based inoculation technology should effectively reduce the use of chemical fertilizers and pesticides in modern agriculture and is considered to be an important strategy for sustainable agriculture. Moreover, more attention should be paid to the relationships among microbes, plants, and soil under natural field conditions, because the successful use of PGPMs in agriculture is usually influenced by the ability of PGPMs to colonize roots, root exudation, and soil health. While microbes isolated from the zone roots of Chinese cabbage plants were highly effective in controlling root rot bacteria, some limitations must be considered when introducing them into a plant disease control management program. These limitations include the extent to which it is affected by environmental factors such as pH, temperature, etc. In addition, the effectiveness of these microbes may be affected by soil types, and there is a possibility that pathogens may develop resistance to them, thus reducing their effectiveness. All of these factors and more must be considered when applying.

## 5 Conclusion

In conclusion, the pathogen of soft rot that caused serious losses on Chinese cabbage in Hangzhou, China, was identified as *P. brasiliense*. Differences in root-zone soil microbial communities were found between the healthy and diseased Chinese cabbage under soft rot stress. Specifically, the bacterial OTU number was less and the fungal OTU number was higher in healthy Chinese cabbage soil than that of diseased ones, whereas there was no significant difference between the diversity of microbial communities. A total of 11 bacterial and 27 fungal biomarker taxa were identified across all healthy and diseased Chinese cabbage soils. The abundances of the phyla Chloroflexi, Bacteroidota, Firmicutes, Ascomycota, Mortierellomycota, and the genus *A4b*, *Mortierella*, and *Podospora* were significantly enriched in healthy Chinese cabbage soils. These microbes might reconstruct the microbial community of Chinese cabbage, provide the first line of defense against soft rot caused by competition or antagonism, and even promote plant growth. A total of 15 bacterial strains were isolated from root-zone soil of diseased Chinese cabbage, and the PGP tests showed the growth promoting effect was particularly obvious by ZT14 (*Citrobacter freundii*), ZT33 (*Enterobacter cloacae*), ZT41 (*Myroides odoratimimus*), ZT52 (*Bacillus paramycoides*), ZT58 (*Klebsiella pasteurii*), ZT45 (*Klebsiella aerogenes*), and ZT32 (*Pseudomonas putida*), but their application effects under field condition need further study. As a result, this study provides new insights into the Chinese cabbage defense mechanisms involved in soft rot stress responses, as well as efficient PGPMs for Chinese cabbage production.

## Data availability statement

The original contributions presented in the study are included in the article/supplementary material, further inquiries can be directed to the corresponding authors.

## Ethics statement

The manuscript presents research on animals that do not require ethical approval for their study.

## Author contributions

XL: Conceptualization, Data curation, Formal analysis, Funding acquisition, Investigation, Methodology, Resources, Software, Supervision, Validation, Visualization, Writing – original draft, Writing – review & editing. XR: Investigation, Writing – original draft. EI: Supervision, Writing – review & editing. HK: Investigation, Writing – original draft. MW: Investigation, Writing – original draft. JX: Investigation, Writing – original draft. HW: Formal analysis, Writing – original draft. LS: Resources, Writing – original draft. TZ: Conceptualization, Validation, Writing – original draft. BL: Project administration, Validation, Writing – review & editing. JY: Conceptualization, Data curation, Formal analysis, Funding acquisition, Investigation, Methodology, Project administration, Resources, Supervision, Validation, Visualization, Writing – original draft, Writing – review & editing.

## Footnotes

^1^https://www.omicstudio.cn/tool/
